# Structural distortions and consequences for magnetism in the 5d^1^ double perovskite Pb_2_MgReO_6_

**DOI:** 10.1039/d6tc01983h

**Published:** 2026-07-28

**Authors:** Hai L. Feng, Martha Greenblatt, Nathan P. Bentley, Tom Lancaster, Adrian D. Hillier, Budhika Mendis, Rebecca Rae, Fabio Orlandi, Emma Suard, Thomas J. Hicken, Emma E. McCabe

**Affiliations:** a Department of Chemistry and Chemical Biology, Rutgers, The State University of New Jersey Piscataway New Jersey 08854 USA; b Beijing National Laboratory for Condensed Matter Physics and Institute of Physics, Chinese Academy of Sciences Beijing 100190 China; c Department of Physics, Durham University Durham DH1 3LE UK emma.mccabe@durham.ac.uk; d ISIS Neutron and Muon Facility, STFC, Rutherford Appleton Laboratory Harwell Campus, Didcot Oxfordshire OX11 0QX UK; e School of Physics and Astronomy, University of Edinburgh Edinburgh EH9 3JZ UK; f Institut Laue-Langevin 71 Avenue des Martyrs 38042 Grenoble France; g PSI Center for Neutron and Muon Sciences 5232 Villigen PSI Switzerland

## Abstract

We report here the structural and magnetic characterisation of the high-pressure double perovskite Pb_2_MgReO_6_. Our analysis using neutron diffraction demonstrates that the second-order Jahn–Teller Pb^2+^ cation plays the dominant structural role, preventing the proposed delicate quadrupolar order from emerging. We explore the relationship between related polar and antipolar structural phases in the context of other complex double perovskites. Muon-spin relaxation (µSR) measurements suggest that a short-range ordered state occurs for 11.3 K < *T* < 30 K, before a collinear AFM state sets in for *T* < 11.3 K. These findings add to our understanding of the structural behaviour of lead-based perovskites, as well as the sensitivity of multipolar order to site symmetry.

## Abstract 2

The crystal structure and phase transitions of 5d^1^ double perovskite Pb_2_MgReO_6_ are investigated using neutron, electron and X-ray diffraction. Magnetisation and μSR measurements give evidence for phases with short-range and long-range magnetic order.

## Graphical abstract

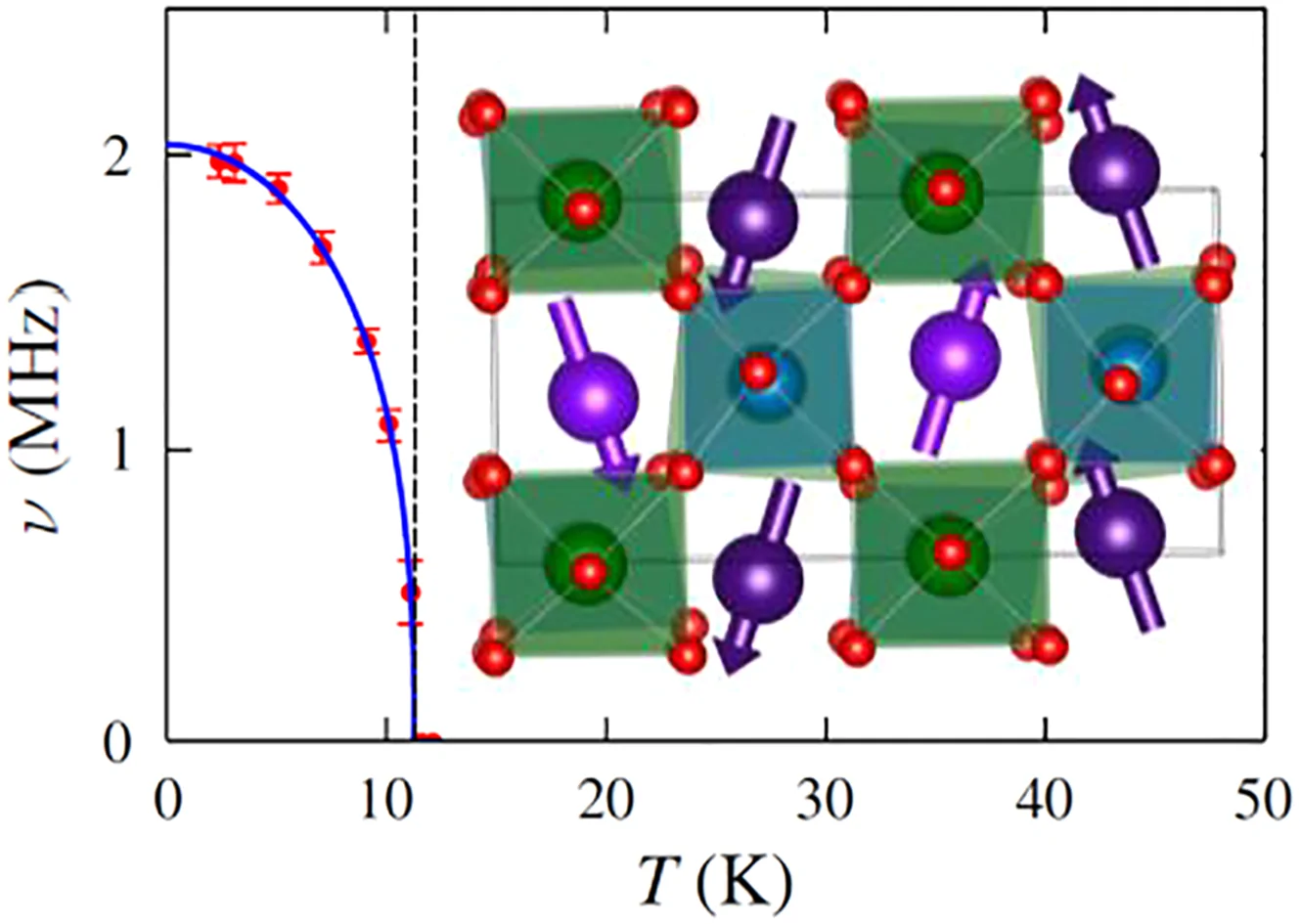

## 1. Introduction

**Fig. 1:**
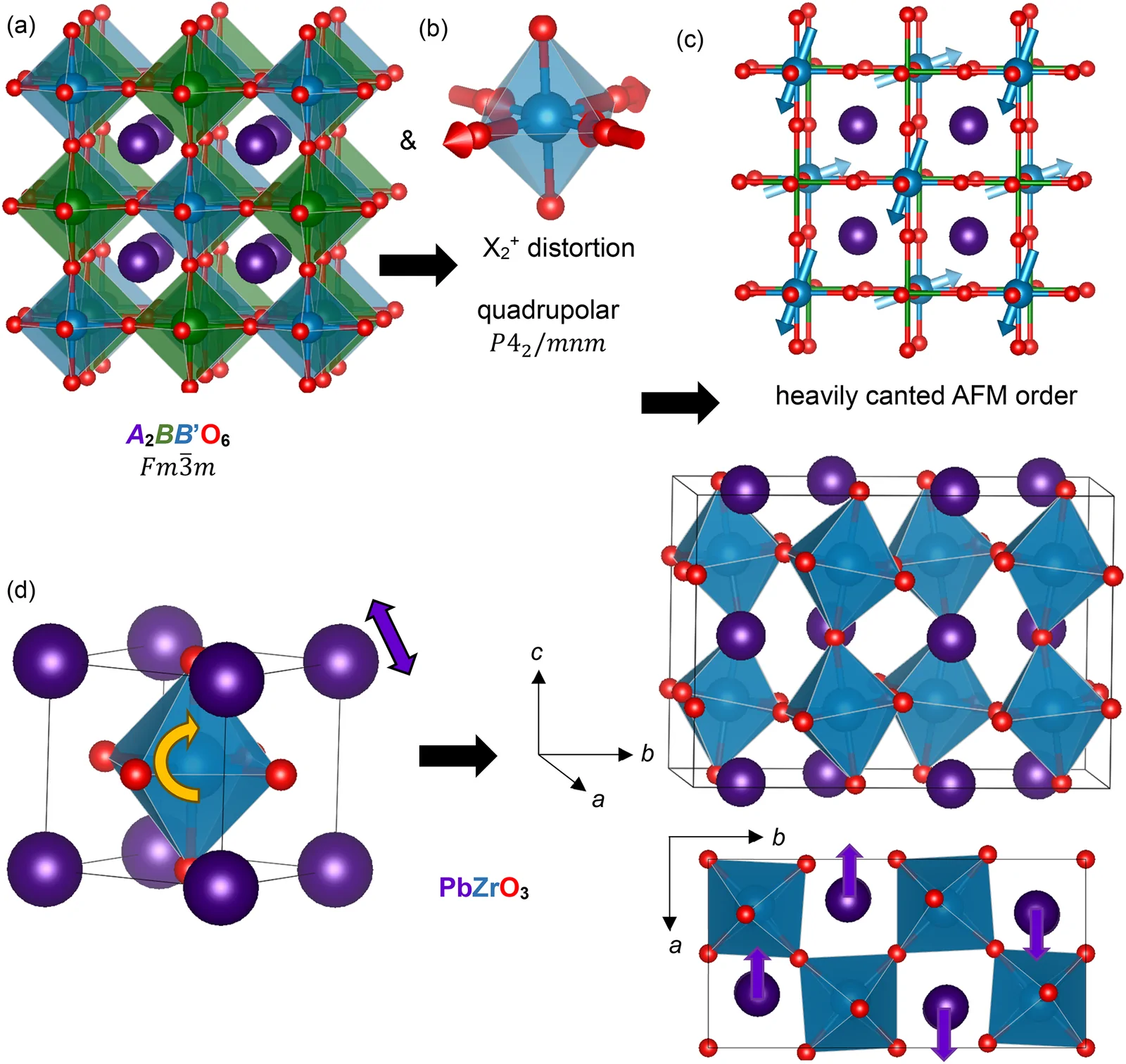
(a) Shows the ideal cubic (*Fm*3̄*m* symmetry) structure of a double perovskite *A*_2_*BB*′O_6_ with *A*, and O ions in purple and red, and *B*O_6_ and *B*′O_6_ corner-linked octahedra in green and blue, respectively; (b) illustrates the X_2_^+^ lattice distortion (O displacements shown by red arrows) that lowers the double perovskite symmetry to *P*4_2_/*mnm* and (c) shows the heavily canted AFM magnetic structure proposed for Ba_2_NaOsO_6_^17^ and Ba_2_MgReO_6_^26^ with Os^7+^/Re^6+^ moments shows by blue arrows; panel (d) highlights the rotations (yellow arrow) of ZrO_6_ octahedra (blue) and Pb^2+^ displacements (purple arrow) on the left and illustrates the large orthorhombic unit cell on the right (with Pb^2+^ displacements highlighted by purple arrows).^31–33^

Materials containing 5d transition metal cations provide an exciting opportunity to explore the delicate interplay between strong spin–orbit coupling and electron correlation, which gives rise to a diversity of magnetic and electronic ground states.^1–6^ Double perovskites *A*_2_*BB*′O_6_,^7^ with rock salt ordering of diamagnetic *B* cations and 5d^1^*B*′ cations (Fig. 1a), are particularly interesting: the separation of *B*′ cations gives sensitivity to the site symmetry and any orbital degeneracy, whilst the radial extent of the 5d orbitals gives strong hybridisation with oxide 2p orbitals, appreciable magnetic exchange interactions and helps favour orbital ordering.^8–12^ Systems such as *A*_2_*B*OsO_6_ (*A* = Ba, Sr; *B* = Li, Na) and *A*_2_MgReO_6_ (*A* = Ca, Sr, Ba) containing the 5d^1^ cations Os^7+^ and Re^6+^ demonstrate both the diversity of ground states and complexity of the terms that stabilise these states.

The synthesis of Ba_2_*B*OsO_6_ (*B* = Li, Na) was first reported in 1962^13^ and later magnetic measurements suggested long-range magnetic order in Ba_2_NaOsO_6_ below 8 K with a small ferromagnetic (FM) component.^14^ Theory and experimental work demonstrated that the small Os^7+^ magnetic moment and the Mott-insulating behaviour of cubic (*Fm*3̄*m* symmetry) Ba_2_NaOsO_6_ resulted from the cooperative effects of spin–orbit coupling (stabilising the *j*_eff_ = 3/2 ground state) and electron correlation.^15,16^ Chen *et al.* highlighted the role of strong hybridisation between Os^7+^ 5d and O 2p orbitals giving rise to a significant electric quadrupole-quadrupole interaction and potentially stabilising an unusual quadrupolar order in Ba_2_NaOsO_6_.^9^ Spectroscopic studies suggested a local symmetry breaking (thought to arise in the quadrupolar phase) at temperatures just above the magnetic ordering temperature,^17^ but attempts to detect signatures of this symmetry breaking in diffraction studies on Ba_2_NaOsO_6_ have not yet been successful.^8^ The low-temperature magnetic state has been described as a “heavily canted AFM”^17^*i.e.* FM alignment of Os^7+^ spins along [11̄0] within layers, and antiparallel alignment from layer to layer to give an overall AFM pattern (described by the mX_5_^+^ magnetic ordering mode, with respect to the parent *Fm*3̄*m* phase with the *B*′ site at the origin, Fig. 1(c)), with a significant FM canting of spins towards the [110] direction (described by mΓ_4_^+^ magnetic ordering mode, again with respect to the *Fm*3̄*m* phase with the *B*′ site at the origin), leading to an FM component along [110], Fig. 1c. Interestingly, neither isostructural Ba_2_LiOsO_6_ nor tetragonal Sr_2_LiOsO_6_ show evidence for quadrupolar order or the heavily-canted AFM state, reflecting the sensitivity towards Os site symmetry and the balance between exchange interactions, anisotropy and electron correlation.^8^

Among the *A*_2_MgReO_6_ double perovskites containing 5d^1^ Re^6+^ cations, the room temperature crystal structure evolves from the ideal cubic structure (*Fm*3̄*m* symmetry) with no rotations of MgO_6_/ReO_6_ octahedra for Ba_2_MgReO_6_,^18–20^ to a tetragonal structure (*I*4/*m* symmetry) for Sr_2_MgReO_6_ with *a*^0^*a*^0^*c*^−^ rotations,^21–23^ to a monoclinic (*P*2_1_/*n* symmetry) structure with *a*^−^*a*^−^*c*^+^ rotations for Ca_2_MgReO_6_,^20^ reflecting the decreasing tolerance factor with decreasing *A* cation size.^7^ This variation in structure changes both the magnetic exchange interactions and the Re^6+^ site symmetry which both play a role in determining the resulting magnetic behaviour: all three *A*_2_MgReO_6_ phases have spin–orbit entangled ground states with *j*_eff_ = 3/2^24^ and although earlier reports suggested that the distorted (frustrated) face-centred cubic lattice of *S* = 1/2 Re^6+^ ions resulted in spin-glass-like behaviour for A_2_MgReO_6_ (A = Ca, Sr),^20–23^ more recent experiments indicate AFM order below *T*_N_ = 55 K for Sr_2_MgReO_6_.^25^

The higher symmetry structure of Ba_2_MgReO_6_, with more isotropic exchange interactions, and higher Re^6+^ site symmetry (*m*3̄*m* in the *Fm*3̄*m* room temperature phase of Ba_2_MgReO_6_, compared with 4/*m* symmetry in Sr_2_MgReO_6_ with static Jahn–Teller distortion), made it a candidate material for quadrupolar order^26^ and indeed, recent spectroscopic work has given evidence for local *P*4_2_/*mnm* lattice distortions and charge quadrupoles occurring simultaneously on cooling below *T*_q_ = 33 K.^27^ On further cooling below *T*_m_ = 18 K^26^ Ba_2_MgReO_6_ orders magnetically, adopting the heavily canted AFM structure with FM component along [110] as described above for Ba_2_NaOsO_6_. Lovesey and Khalyavin explored the coupling between the lattice distortion (described by the X_2_^+^ irrep)^28–30^ and the magnetic order, and, consistent with work by Cruz Pinha Barbosa,^30^ showed that the heavily canted AFM phase arises from the orbital-ordering driven distortion: the X_2_^+^ lattice distortion (irrep with respect to the parent *Fm*3̄*m* phase with the *B*′ site at the origin, describing alternating compression/elongation and of each ReO_6_ octahedra within the *ab* plane, (Fig. 1b) combined with mX_5_^+^ AFM order, by symmetry gives rise to the FM mΓ_4_^+^ component^29^ and hence the heavily canted AFM magnetic structure, Fig. 1c. This explains the sensitivity of these systems to slight changes in Re orbital occupation and site symmetry which can easily be disrupted, preventing quadrupolar order.

In addition to the geometric role played by *A* cations of different sizes relative to the *B*/*B*′ cations, structural distortions can also result from electronic factors including the second-order Jahn–Teller (SOJT) effect often observed for 6s^2^ cations such as Pb^2+^ and Bi^3+^.^34^ The perovskite PbZrO_3_ provides a good illustration of this, with the ground state structure allowing both rotations of ZrO_6_ octahedra (*a*^−^*a*^−^*c*^0^) as well as displacements of Pb^2+^ cations, Fig. 1d.^31–33^ The Pb^2+^ displacements are described by the Σ_2_ mode with *k* vector (¼ ¼ 0) (with respect to the simple cubic perovskite of *Pm*3̄*m* symmetry, with *B* site in the origin) which, when combined with ZrO_6_ tilts, gives a large orthorhombic cell with dimensions *a* = 2*a*_p_, 
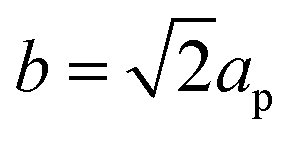
 and 
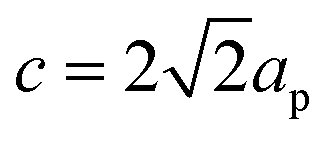
 (*a*_p_ denotes the unit cell parameter of the parent cubic *Pm*3̄*m* perovskite).^31,32^ Closely related structures have been reported for several Pb_2_*BB*′O_6_ double perovskites including Pb_2_MnWO_6_,^35–37^ Pb_2_MgWO_6_^38^ and Pb_2_MnReO_6_.^39^ Due to the *B*/*B*′ cation order in these double perovskites, the Pb^2+^ displacements are described by Σ_2_ mode with *k* vector (½ ½ 0) (or (⅔ ⅔ 0) for Pb_2_MnReO_6_)^39^ relative to the parent *Fm*3̄*m* phase but as for PbZrO_3_, this again gives the *a* = 2*a*_p_, 
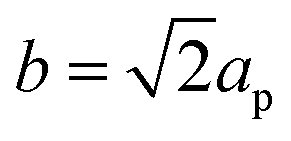
 and 
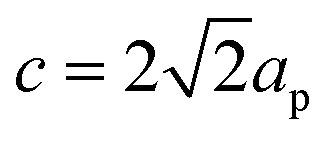
 sized orthorhombic unit cell^35,38^ (or closely-related monoclinic cell for Pb_2_MnReO_6_).^39^ In contrast to PbZrO_3_, the *B*O_6_/*B*′O_6_ octahedra in the double perovskites do not behave as rigid units and no clear pattern of octahedral tilts is observed.^38^ However surprisingly the double perovskite Pb_2_CoTeO_6_ behaves quite differently, with the SOJT of the Pb^2+^ cation being less dominant and a series of tilt transitions are reported on cooling,^40^ despite the tolerance factor for Pb_2_CoTeO_6_ being comparable to those of Pb_2_MnWO_6_, Pb_2_MgWO_6_, Pb_2_MnReO_6_ and Pb_2_MgReO_6_ discussed in this work.

We report here the structural and magnetic characterisation of the high-pressure high-temperature synthesized double perovskite Pb_2_MgReO_6_. Our analysis using neutron diffraction demonstrates that the SOJT Pb^2+^ cation plays the dominant structural role, preventing the delicate quadrupolar from emerging. Muon spin relaxation (µSR) measurements suggest that a spin-glass-like phase occurs for 11.3 K < *T* < 30 K before a collinear AFM state is observed for *T* < 11.3 K.

## 2. Methods

Pb_2_MgReO_6_ was synthesised by solid state reactions from powders of PbO (99.99%, Alfa Aesar), MgO (99.99%, Alfa Aesar) and ReO_3_ (99.9%, Alfa Aesar). ReO_3_ was annealed at 473 K overnight before use because it is mildly hygroscopic. A molar ratio 2 : 1 : 1 of reagents PbO : MgO : ReO_3_ were thoroughly ground, and sealed in a Pt capsule, which was placed in an MgO crucible. The crucible was then statically compressed in a Walker-type multianvil press^41^ at a pressure of 6 GPa. After heating at 1273 K for 1 hour while maintaining high-pressure conditions, the sample was then quenched to ambient temperature. The pressure was then gradually released over several hours. A dense, black polycrystalline pellet was obtained.

The temperature dependence of the specific heat capacity (*C*_p_) was measured using a Physical Properties Measurement System (Quantum Design, Inc.) by a thermal relaxation method at temperatures between 2 and 300 K with Apiezon N grease thermally connecting the material to the holder stage. The magnetic susceptibility *χ* of a Pb_2_MgReO_6_ powder sample was measured in a Magnetic Properties Measurement System (Quantum Design, Inc.). The measurement was conducted in field-cooled (FC) and zero-FC conditions in the temperature range between 2 and 300 K. The applied magnetic field was 1 kOe. Data were corrected for the diamagnetic contribution of core electrons.

Selected area electron diffraction (SAED) data were collected using a Jeol 2100F transmission electron microscope operating at 200 keV. The sample was deposited onto a holey carbon grid. This was mounted in a double-tilt sample holder, and zone-axis diffraction patterns were acquired using a Gatan Rio camera. Energy dispersive X-ray (EDX) data were collected on the crystallites used for SAED using an Oxford Instruments X-Max 65T silicon drift detector.

Variable temperature high resolution powder X-ray diffraction (XRPD) data were collected at University of Edinburgh using a Rigaku Smartlab diffractometer equipped with a Johansson monochromator combined with Cross Beam Optics (CBO)^42^ parabolic mirrors, with an Oxford Cryosystems Phenix sample stage. A thin layer of grease was added to the surface of a Cr-plated copper sample holder and the Pb_2_MgReO_6_ sample was sprinkled on top. The sample was cooled to 12 K and a high-quality data set (18 hours) was collected (5–100° 2*θ*, step size 0.5° minute^−1^). The sample was then warmed to 300 K with data (20 minute scans, 15–80° 2*θ*, 3.5° minute^−1^) collected at 5 K intervals (with 10 minutes equilibration before each scan).

Neutron powder diffraction (NPD) data were collected on a 479.9 mg Pb_2_MgReO_6_ powder sample placed inside a 3 mm diameter thin-walled cylindrical vanadium can on the WISH diffractometer at the STFC ISIS Neutron and Muon Source (Rutherford Appleton Laboratory, U.K.) located in the second target station.^32^ Multibank (average two-theta of 153°, 122°, 90°, 58°, 27° each covering 32 degrees of the scattering plane) data were collected at 250 K (8 hours) and the sample was then cooled to 1.5 K in a ^4^He cryostat and a high quality dataset (8 hours) was recorded. High resolution low temperature NPD data were also collected on the D2B diffractometer (with Ge monochromator to give *λ* = 1.594 Å) at the Institut Laue Langevin (ILL) (Grenoble). The same sample was placed in 5 mm diameter vanadium can cooled to 3.5 K and multiple scans collected over 10 hours were summed to give a high-quality data set. The sample was then warmed to 17 K and again multiple scans collected over 8 hours were summed to give a second high quality data set. All powder diffraction data were analysed by the Rietveld method^43^ using TopasAcademic software^44,45^ and local subroutines. Due to the challenges associated with calculating standard uncertainties in refined parameters, we take a conservative approach and the uncertainties quoted here are three times those quoted by the Rietveld software.^46,47^ Symmetry analysis was carried out using the web-based ISODISTORT suite of programmes.^29,48^

We performed zero-field (ZF), longitudinal field (LF) and weak transverse field (wTF) muon-spin relaxation (µSR) measurements^49^ using the EMU spectrometer^50^ at the STFC ISIS Neutron and Muon source, Rutherford Appleton Laboratory, UK, with some supporting measurements made at SµS (Paul Scherrer Institute, Switzerland), using the GPS instrument.^51^ For these measurements, a powder sample of Pb_2_MgReO_6_ (∼400 mg) was packed in a silver foil envelope (foil thickness 25 µm), resulting in a random orientation of crystallites relative to the initial spin direction of the muon. The small size of the sample mandated the use of the flypast geometry,^52^ where the sample is suspended on a fork in a ^4^He cryostat. Candidate muon stopping sites were determined using the MuFinder program,^53^ which is based on the CASTEP^54^ plane-wave pseudo-potential code. Muonated supercells consisting of a muon (modelled by an ultra-soft hydrogen pseudopotential) placed in a 2 × 2 × 1 supercell of Pb_2_MgReO_6_ in the low-temperature orthorhombic phase (*Pmc*2_1_ symmetry) were generated, with muons constrained to be at least 1.0 Å away from atoms and at least 0.5 Å away from muons in other supercells. 30 of these muonated supercells were then relaxed using the PBE functional,^55^ a plane-wave cutoff energy of 1150 eV and a 2 × 2 × 2 Monkhorst–Pack grid^56^ for the Brillouin zone sampling, giving total energies converged to within 10 meV per atom.

## 3. Results

### 3.1. Heat capacity measurements

**Fig. 2:**
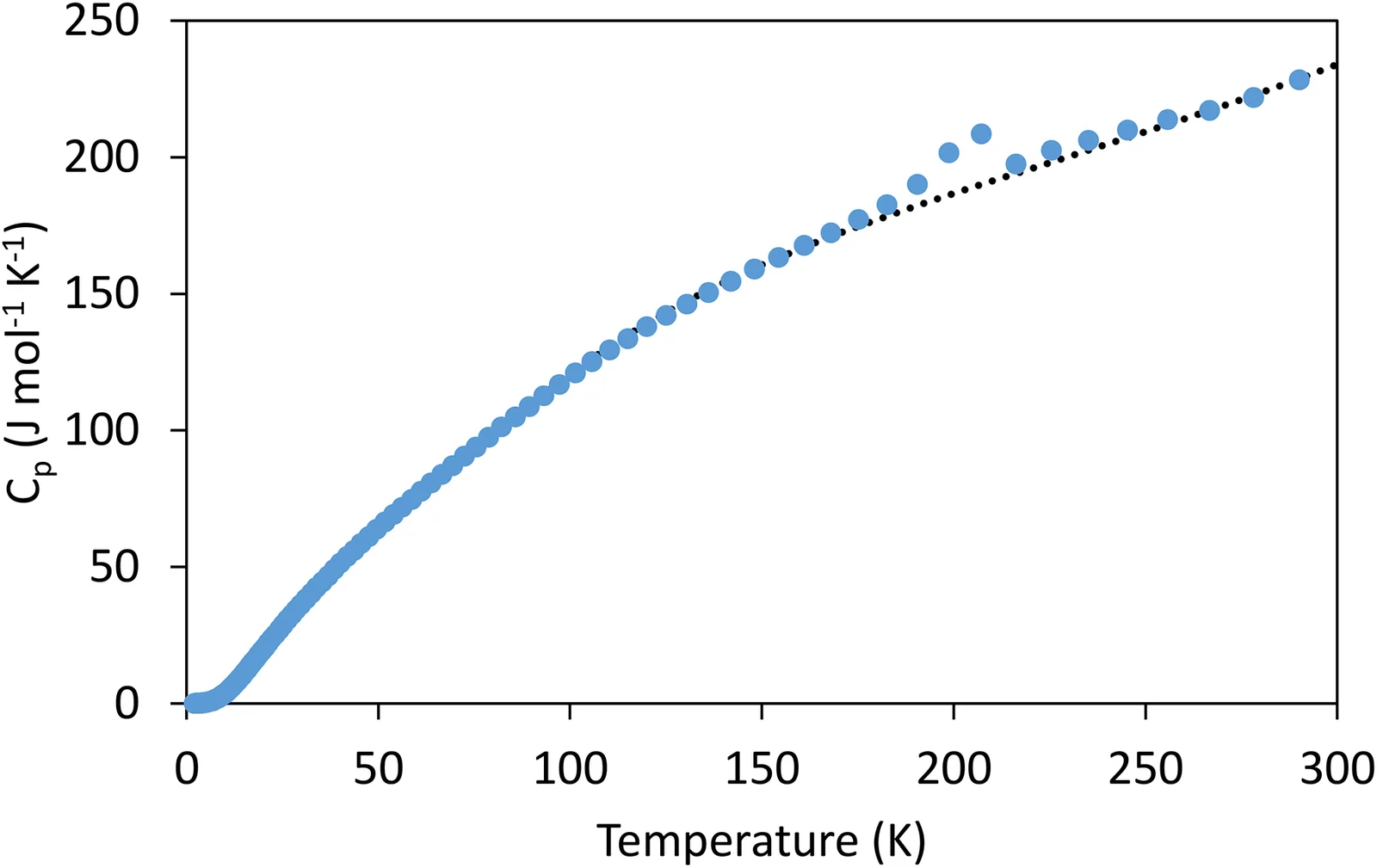
Heat capacity measurements for Pb_2_MgReO_6_ showing structural phase transition at 207 K. Filled points show experimental data and dashed line shows fit to an Einstein–Debye model with two Einstein terms *(Θ*_D_ ≈ 250 K, *Θ*_E1_ ≈ 350 K and *Θ*_E2_ ≈ 84 K).

The heat capacity of Pb_2_MgReO_6_ was measured on warming (Fig. 2), indicating a phase transition at ∼200 K. The lattice contribution to the heat capacity could be approximately fitted by a Debye–Einstein model with two Einstein terms,^57^ with Debye temperature *Θ*_D_ ∼ 250 K and Einstein temperatures *Θ*_E1_ and *Θ*_E2_ ∼350 K and 84 K. The entropy associated with the phase transition is estimated at ∼0.2 J mol^−1^ K^−1^. There is no evidence in these heat capacity data for the low temperature phase transition revealed by µSR measurements (see Section 3.5).

### 3.2. High temperature structural characterisation

**Fig. 3:**
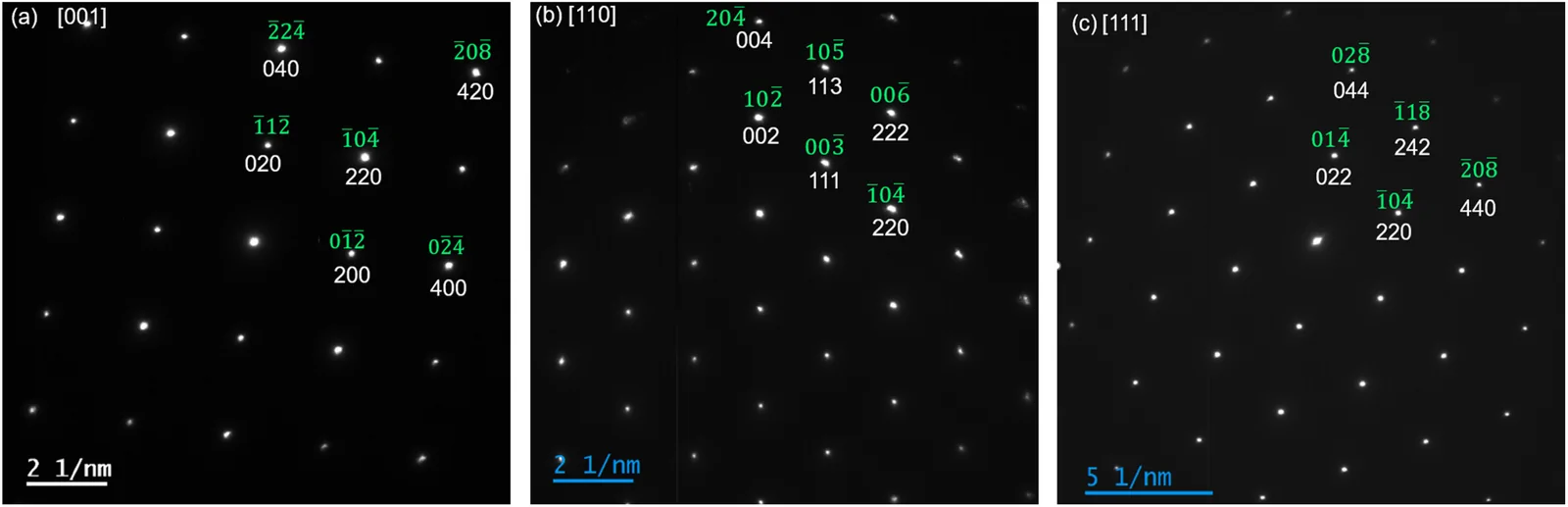
Selected-area electron diffraction patterns for (a) [001] zone axis, (b) [110] zone axis and (c) [111] zone axis taken at room temperature for Pb_2_MgReO_6_. Indices are given in white for the *Fm*3̄*m* unit cell and in green for the *R*3̄ unit cell (using a hexagonal cell setting).

Room temperature selected area electron diffraction patterns for Pb_2_MgReO_6_ are shown in Fig. 3. All diffraction spots could be indexed by the *B*-site ordered double perovskite model with *Fm*3̄*m* symmetry but an angle ∼87° between *hh*0 and 00*l* reflections (Fig. 3b) in the [110] zone axis led us to consider a rhombohedral model of *R*3̄ symmetry.

**Fig. 4:**
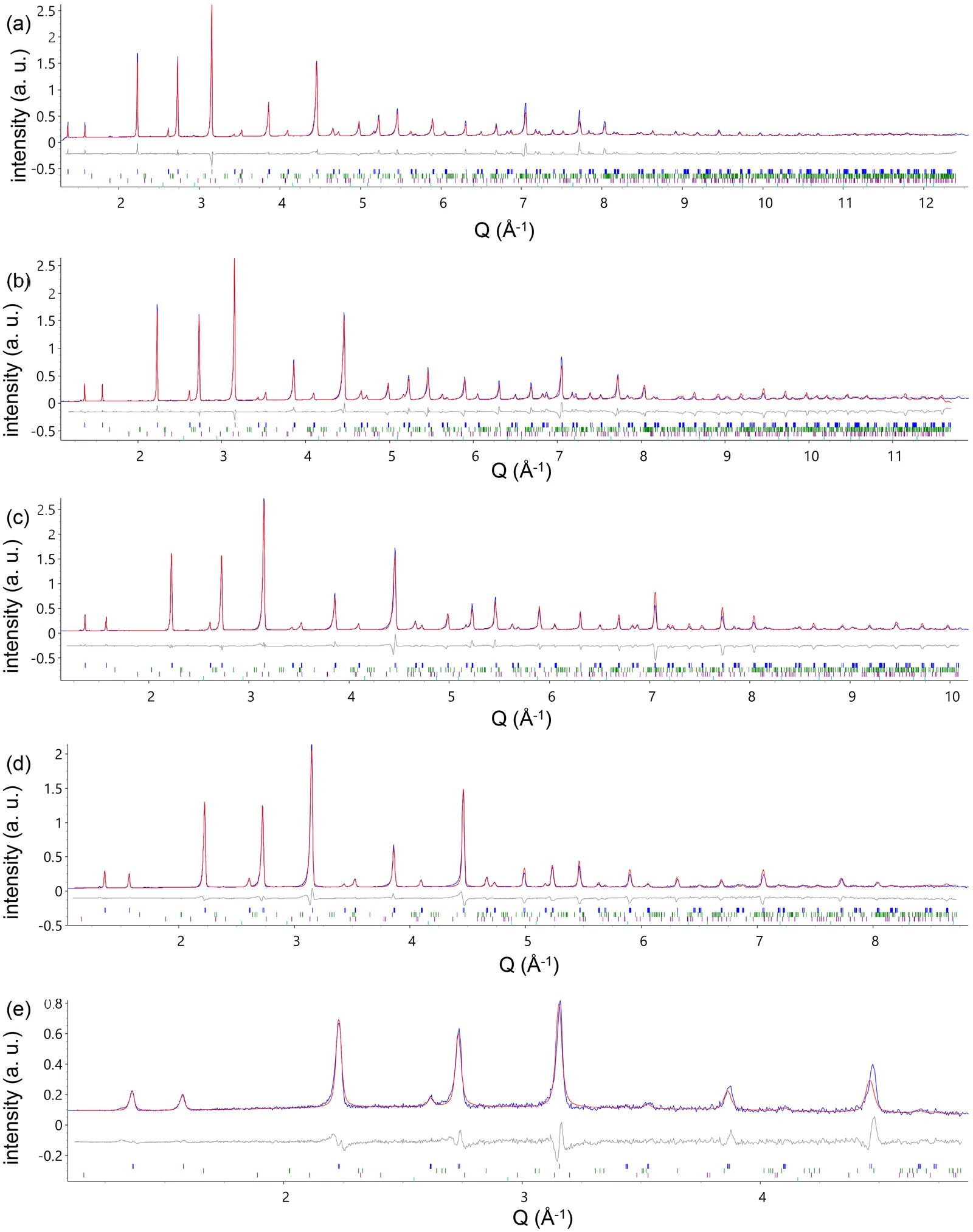
Rietveld refinement profiles for 250 K NPD data for Pb_2_MgReO_6_ using a rhombohedral model of *R*3̄ symmetry showing (a) bank 5_6 (153°), (b) bank 4_7 (122°), (c) bank 3_8 (90°), (d) bank 2_9 (58°) and (e) bank 1_10 (27°) banks of data. Observed, calculated and difference profiles are shown in blue, red and grey, respectively; *R*_wp_ = 6.12%, *R*_p_ = 6.20% and *χ*^2^ = 10.54. Tick marks show peak positions for main phase (top blue (97(1)% by weight), PbO_2_ (upper middle green, 0.8(1)% by weight), PbO (lower middle purple, 0.61(7)% by weight) and MgO (bottom cyan, 2(1)% by weight).

Neutron powder diffraction (NPD) data collected at 250 K were also initially fitted using a cubic model of *Fm*3̄*m* symmetry but a slight splitting of *hhh* reflections was observed, consistent with a rhombohedral distortion (to give *h*0*l* and 00*l* reflections of the rhombohedral model, assuming a hexagonal cell setting), see SI. The data were fitted well by a model of *R*3̄ symmetry, similar to that reported for Pb_2_CoTeO_6_ (210 ≤ *T* ≤ 370 K)^40^ and for Pb_2_CoReO_6_.^1^ This model allows equal rotations of the *B*O_6_ octahedra about each of the axes of the parent *Pm*3̄*m* perovskite structure (*a*^−^*a*^−^*a*^−^ tilts, described by the R_4_^+^ irrep with respect to the parent *Pm*3̄*m* perovskite,^58^ or Γ_4_^+^ with respect to the un-tilted *B*-cation-ordered *Fm*3̄*m* double perovskite).^29^ Final structural details and refinement profiles are given in Table 1 and Fig. 4. Refining the degree of order of Mg^2+^ and Re^6+^ cations over the two *B* sites revealed a high degree of ordering (93(1)% ordered) and a fully cation-ordered model was used for all subsequent analysis.

**Table 1:** Details from Rietveld refinement using 250 K NPD data for Pb_2_MgReO_6_ using a rhombohedral model of *R*3̄ symmetry; *a* = *b* = 5.6342(12) Å, *c* = 13.7703(3) Å, volume = 378.73(2) Å^3^

Atom	Wyckoff site	*x*	*y*	*z*	*U*_iso_ × 100 (Å^2^)
Pb	6c	0	0	0.2498 (12)	3.17 (6)
Mg	3b	0	0	0.5	1.0 (3)
Re	3a	0	0	0	1.06 (18)
O	18f	0.1875 (6)	−0.1330 (9)	0.0797 (6)	2.59 (6)

Other lower symmetry models (including those of *P*2_1_/*n* symmetry (allowing *a*^−^*a*^−^*c*^+^ tilts as reported for Ca_2_MgReO_6_), *I*4/*m* symmetry (allowing *a*^0^*a*^0^*c*^+^ tilts as reported for Sr_2_MgReO_6_) and *I*2/*m* symmetry (allowing *a*^0^*b*^−^*b*^−^ tilts as reported for Pb_2_CoTeO_6_) were also considered but these gave worse fits to the data. To estimate the stoichiometry of the sample, this *R*3̄ model was refined to fit the high resolution data (bank 5_6, 2*θ* = 153°), using a single global atomic displacement parameter, and refining the occupancies of Pb, Re and O sites, while the Mg site occupancy was fixed at 1. This refined to give a sample stoichiometry of Pb_1.88(2)_MgRe_1.02(2)_O_5.57(5)_. This is close to the target stoichiometry, but the oxygen vacancies will result in some reduction of Pb and Re ions. Without accurate room temperature bond lengths (*e.g.* from analysis of room temperature NPD data), it is hard to calculate reliable bond valence sum values (these were derived for room temperature structures). Using the 250 K bond lengths, we estimate valences of 2.1 for Pb and Mg sites^59^ (consistent with the expected valences of +2, given the slight contraction on cooling from room temperature to 250 K). This would suggest that lead is present in +2 oxidation state, and it is unlikely there is appreciate Pb^4+^ content (in contrast to the high pressure perovskites PbCoO_3_^60^ and PbNiO_3_^61,62^). Bond valence parameters for Re^6+^ are not available but calculations using parameters for Re^5+^ and Re^7+ ^^59^ give apparent valences of 5.1 and 6.3 *i.e.* Re^7+^ would be underbonded, and the site seems consistent with Re^5+^. The stoichiometry estimated from refinement, Pb_1.88(2)_MgRe_1.02(2)_O_5.57(5)_, implies Re^5.4+^ (assuming Pb^2+^), indicating a mix of Re^5+^ and Re^6+^ present in our sample.

### 3.3. Variable temperature structural characterisation

**Fig. 5:**
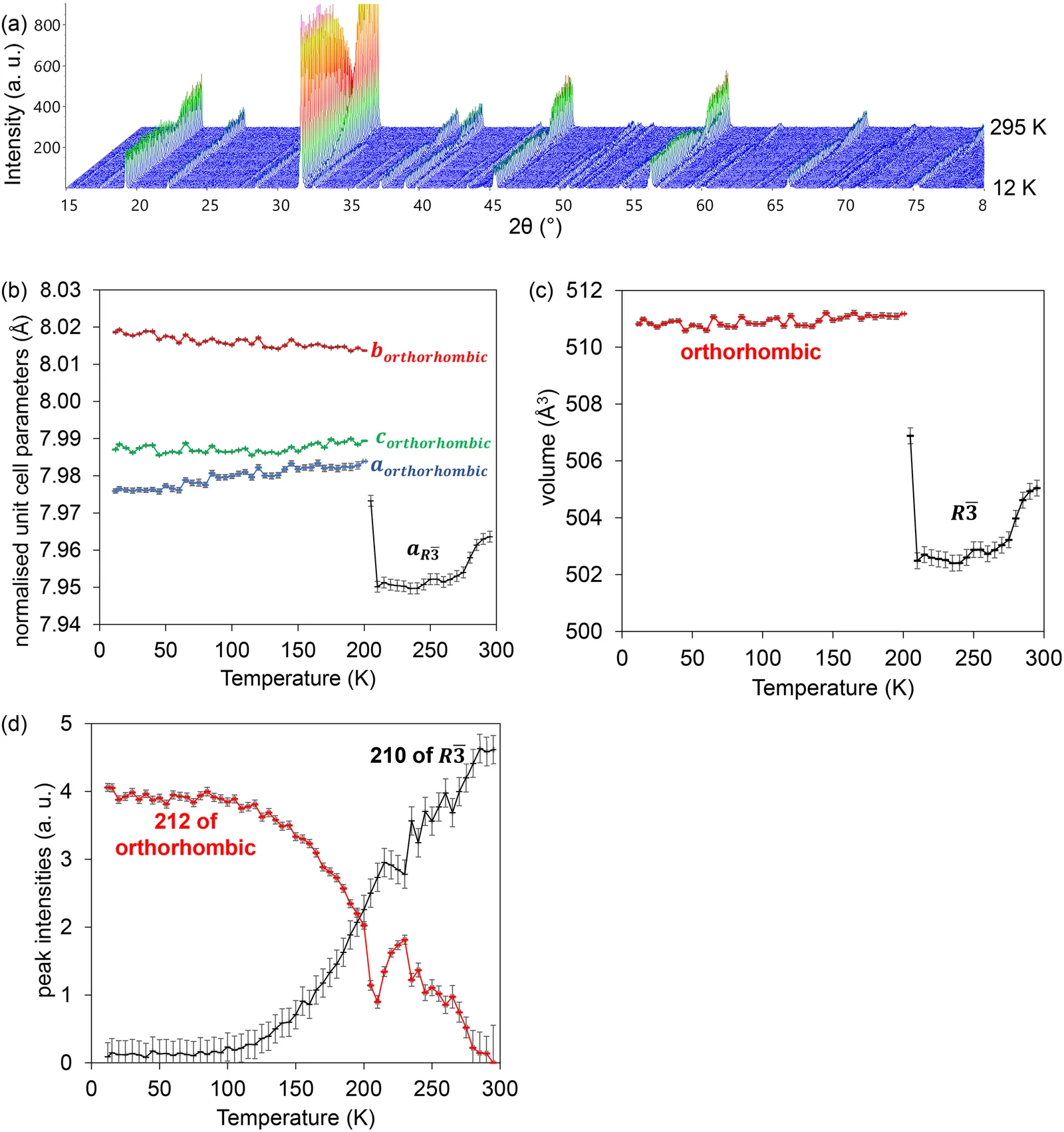
(a) Shows variable temperature XRPD data for Pb_2_MgReO_6_; lower panels show results from Pawley refinements using the *R*3̄ phase and a *P*222 phase to model the high- and low-temperature phases, respectively, showing (b) unit cell parameters (normalised to those of the untilted *Fm*3̄*m B*-site ordered double perovskite model) with *a*_*R*3̄_ in black, and *a*_ortho_, *b*_ortho_ and *c*_ortho_ shown in blue, red and green, respectively; (c) shows unit cell volumes of the *R*3̄ phase (black) and *P*222 phase (red) and (d) shows the intensity of the most intense peaks of the *R*3̄ phase (210, black) and *P*222 phase (212, red).

X-ray powder diffraction (XRPD) data were collected on warming from 12 K to 295 K and analysis gave clear evidence of a structural phase transition at 200 – 210 K (Fig. 5). Additional peaks were observed below this temperature, indicating that a much larger orthorhombic unit cell (*a* = 2*a*_p_, 
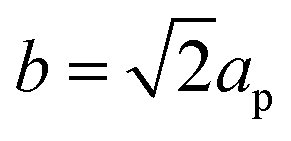
 and 
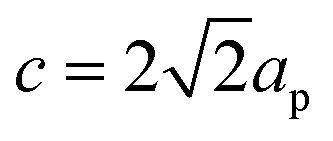
) is required to describe the low temperature structure. Peaks due to both the *R*3̄ and the orthorhombic phase are observed for a wide temperature range (traces of the *R*3̄ phase are observed from at least 105 K, and traces of the orthorhombic phase can still be observed up to at least 245 K). Sequential Pawley refinements indicate an abrupt change in unit cell volume at the transition temperature, consistent with a first-order phase transition (Fig. 5).

### 3.4. Low temperature structural characterisation

Neutron powder diffraction (NPD) data collected at 17 K and at 3.5 K (on the high resolution D2B diffractometer at ILL) indicated many additional peaks not fitted by the high temperature *R*3̄ model (see SI), and instead consistent with a primitive orthorhombic cell, with transformation matrix {(1 1 0) (0 0 1) (½ −½ 0)} relative to the parent *Fm*3̄*m* model. ISODISTORT^29,48^ was used for symmetry analysis (see SI) and possible low symmetry models of *Pnma* and *Pmc*2_1_ symmetries were identified, related to those reported for Pb_2_MnWO_6_,^35^ Pb_2_MgWO_6_,^38,63^ Pb_2_CdWO_6_ and Pb_2_YTaO_6_.^64^ (A *Pna*2_1_ model (as also reported for Pb_2_MnWO_6_)^36^ was also tested but did not give such a good fit to the data for a comparable number of parameters (see SI).) A hallmark of these *Pnma* and *Pmc*2_1_ structures is the displacement of Pb^2+^ cations described by the Σ_2_ irrep (*k* = 1/2 1/2 0) (with respect to the parent *Fm*3̄*m* model). This distortion can give models of *Pnma*, *Pmma* or *Pmc*2_1_ symmetries. This distortion can be broken down into two modes in our test model (see SI). The main difference between these models is as follows:

• *Pnma* model: this distortion acts on all Pb^2+^ sites to give displacements of equal magnitude but half displaced in the positive direction, half in the negative direction (similar to the lower panel of Fig. 1d) to give an antipolar phase (both Σ_2_ modes are constrained to be equal in amplitude);

• *Pmma* model: this antipolar distortion only acts on half the Pb^2+^ sites (only one of the Σ_2_ modes has non-zero amplitude);

• *Pmc*2_1_ model: the distortion acts on all Pb^2+^ sites but displacements in the positive and negative directions have different magnitudes, giving an overall polarisation (both Σ_2_ modes have non-zero amplitude, but different magnitudes).

**Fig. 6:**
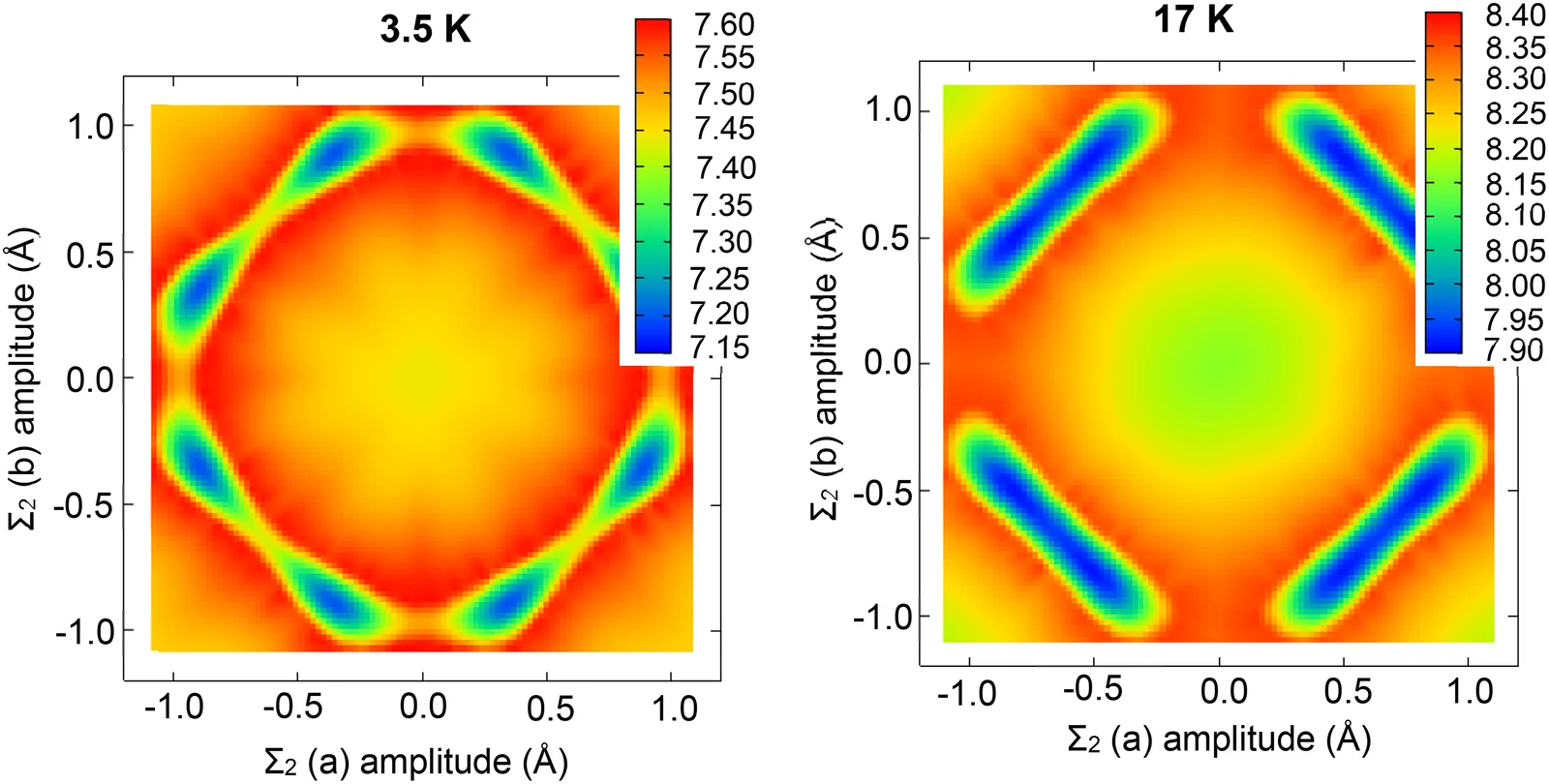
Surface plot showing fit (measured by *R*_wp_) from Rietveld refinements using constant-wavelength NPD data collected at 3.5 K and at 17 K for different amplitudes of the two Pb^2+^ Σ_2_ displacive modes. The plots indicate that the best fit is obtained at both temperatures with non-zero amplitudes for both modes (indicating that both are active). The 3.5 K data is fitted best by models with unequal mode amplitudes, consistent with models of *Pmc*2_1_ symmetry.

Fig. 6 gives surface plots showing the fit to the observed data (measured by *R*_wp_) as a function of the amplitudes of these two Σ_2_ modes for NPD data collected at 3.5 K and at 17 K. At both temperatures, the best fits are found for non-zero amplitudes of both modes, ruling out the *Pmma* model. At 3.5 K, the best fits are found for models with a clear difference in amplitudes for the two modes, indicating greater deviation from the *Pnma* model towards the polar *Pmc*2_1_ model. This supports our assignment of this polar space group for the low-temperature structure. At 17 K, rather than observing a strict 1 : 1 correlation between the amplitudes of the two modes (*e.g.* Σ_2_Pb(1)_ = Σ_2_Pb(2)_ as expected for a *Pnma* model), the mode amplitudes are correlated but are not necessarily of equal magnitudes. This may indicate a reduction of the polar distortion (associated with *Pmc*2_1_ symmetry rather than *Pnma*) with increasing temperature.

**Fig. 7:**
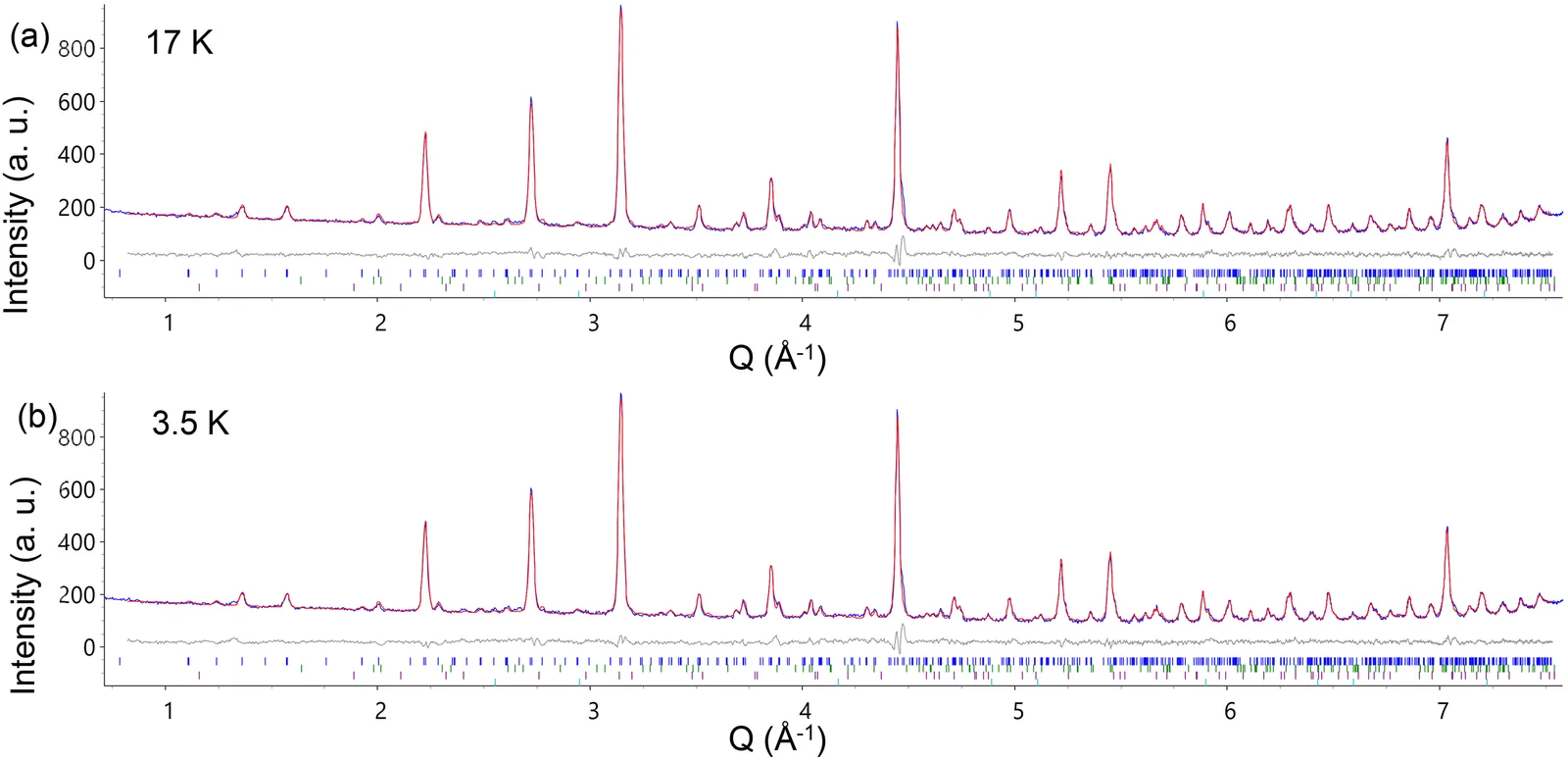
Rietveld refinement profiles showing fits to *Pmc*2_1_ models for Pb_2_MgReO_6_ at (a) 17 K and (b) 3.5 K; observed, calculated and difference profiles are shown in blue, red and grey, respectively and top (blue), upper (green), lower (purple) and bottom (cyan) ticks show peak positions of main phase (98(9)% by weight), PbO_2_ (1.2(6)% by weight), PbO (0(9)% by weight) and MgO (1.1(9)% by weight).

**Fig. 8:**
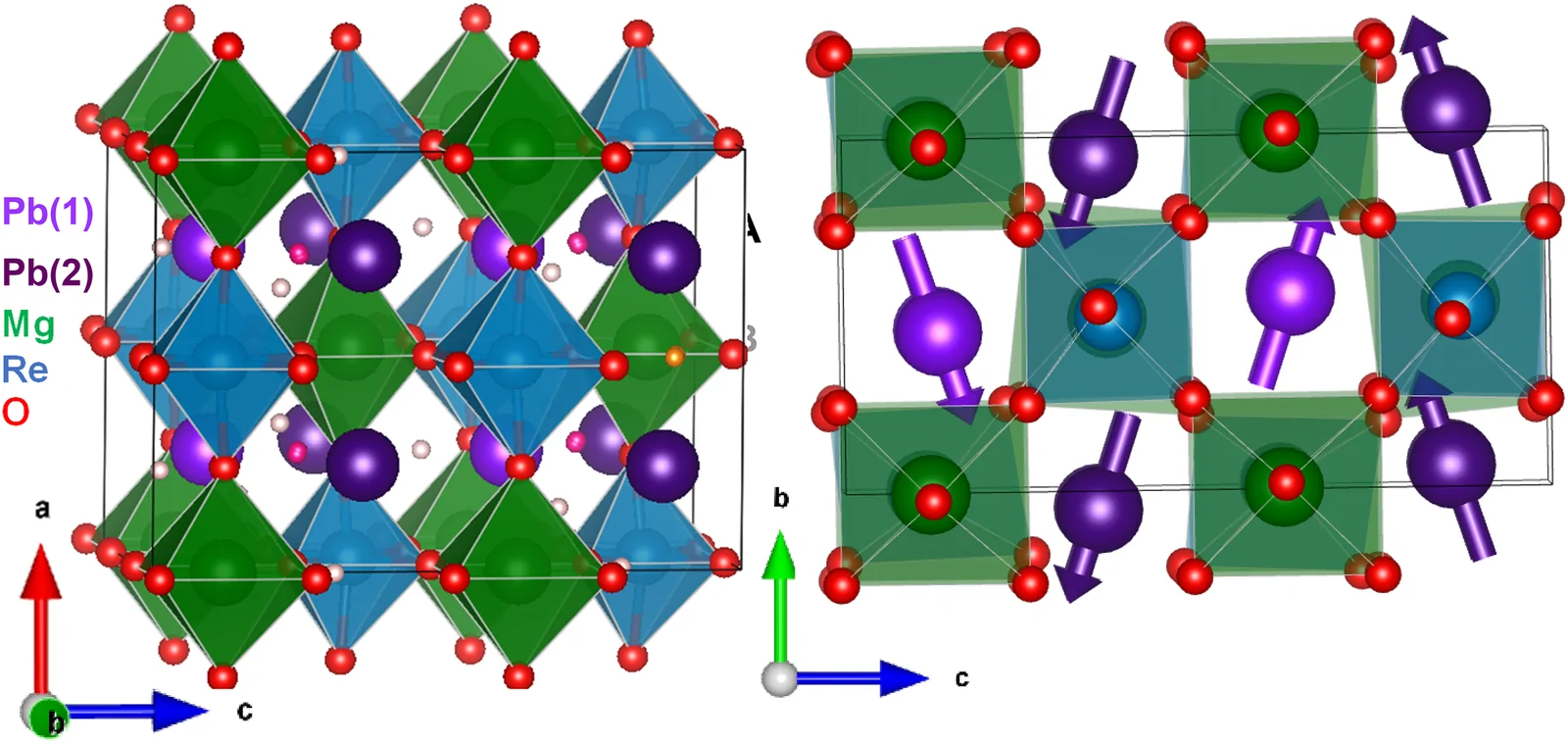
Pb_2_MgReO_6_ structure of *Pmc*2_1_ symmetry refined from 3.5 K NPD data showing MgO_6_ and ReO_6_ octahedra in green and blue, and O and Pb sites in red and purple; displacements of Pb(1) and Pb(2) sites are shown by light and dark purple arrows, respectively. In the left panel, calculated muon stopping sites (Table S4) are shown as small pink spheres with those in groups A and B highlighted in bright pink and orange, respectively.

The *Pmc*2_1_ model gives a significantly better fit at both 17 K and at 3.5 K, with similar amplitudes for the in-plane polar mode refined at both temperatures (mode amplitude of 0.79 Å at 3.5 K compared with 0.80 Å at 17 K). Refinement details are given in Table 2, refinement profiles are shown in Fig. 7 and the 3.5 K structure illustrated in Fig. 8.

**Table 2:** Rietveld refinement details for refinements using *Pmc*2_1_ models for Pb_2_MgReO_6_ for NPD data collected at 3.5 K and 17 K showing refined coordinates

		3.5 K	17 K
	*a* (Å)	7.9805 (3)	7.9805 (3)
	*b* (Å)	5.6648 (3)	5.6650 (3)
	*c* (Å)	11.2965 (9)	11.2966 (9)
	Volume (Å^3^)	510.69 (6)	510.72 (6)
Pb(1) 4c	*x*	0.742 (3)	0.741 (3)
	*y*	0.450 (3)	0.450 (3)
	*z*	0.136 (6)	0.1366 (18)
	*U*_iso_ × 100 (Å^2^)	0.1 (2)	0.1 (2)
Pb(2) 4c	*x*	0.251 (3)	0.251 (3)
	*y*	0.056 (3)	0.056 (3)
	*z*	0.860 (6)	0.860 (2)
	*U*_iso_ × 100 (Å^2^)	0.1 (2)	0.1 (2)
Mg(1) 2b	*y*	0.481 (15)	0.482 (15)
	*z*	0.376 (12)	0.374 (9)
	*U*_iso_ × 100 (Å^2^)	0.02 (6)	0.01 (6)
Mg(2) 2a	*y*	0.004 (15)	0.005 (15)
	*z*	0.620 (9)	0.619 (9)
	*U*_iso_ × 100 (Å^2^)	0.02 (6)	0.01 (6)
Re(1) 2b	*y*	−0.010 (9)	−0.010 (9)
	*z*	0.112 (9)	0.112 (3)
	*U*_iso_ × 100 (Å^2^)	0.02 (3)	0.03 (3)
Re(2) 2a	*y*	0.509 (9)	0.507 (9)
	*z*	0.874 (9)	0.873 (6)
	*U*_iso_ × 100 (Å^2^)	0.02 (3)	0.03 (3)
O(1) 4c	*x*	0.250 (6)	0.248 (6)
	*y*	−0.020 (3)	−0.020 (3)
	*z*	0.122 (9)	0.122 (3)
	*U*_iso_ × 100 (Å^2^)	0.08 (18)	0.06 (18)
O(2) 4c	*x*	0.760 (6)	0.758 (6)
	*y*	0.476 (3)	0.474 (3)
	*z*	0.860 (9)	0.860 (3)
	*U*_iso_ × 100 (Å^2^)	0.08 (18)	0.06 (18)
O(3) 2b	*y*	0.222 (12)	0.219 (9)
	*z*	−0.019 (9)	−0.019 (6)
	*U*_iso_ × 100 (Å^2^)	0.08 (18)	0.06 (18)
O(4) 2a	*y*	0.25 (2)	0.25 (2)
	*z*	−0.008 (12)	−0.007 (9)
	*U*_iso_ × 100 (Å^2^)	0.08 (18)	0.06 (18)
O(5) 2b	*y*	0.797 (9)	0.797 (9)
	*z*	0^a^	0^a^
	*U*_iso_ × 100 (Å^2^)	0.08 (18)	0.06 (18)
O(6) 2a	*y*	0.743 (18)	0.743 (18)
	*z*	−0.008 (9)	−0.009 (9)
	*U*_iso_ × 100 (Å^2^)	0.08 (18)	0.06 (18)
O(7) 2b	*y*	0.243 (12)	0.241 (12)
	*z*	0.232 (9)	0.232 (9)
	*U*_iso_ × 100 (Å^2^)	0.08 (18)	0.06 (18)
O(8) 2a	*y*	0.259 (15)	0.259 (15)
	*z*	0.759 (9)	0.759 (6)
	*U*_iso_ × 100 (Å^2^)	0.08 (18)	0.06 (18)
O(9) 2b	*y*	0.816 (9)	0.816 (9)
	*z*	0.265 (9)	0.265 (6)
	*U*_iso_ × 100 (Å^2^)	0.08 (18)	0.06 (18)
O(10) 2a	*y*	0.753 (15)	0.752 (12)
	*z*	0.758 (9)	0.759 (6)
	*U*_iso_ × 100 (Å^2^)	0.08 (18)	0.06 (18)
	*R*_wp_ (%)	4.17	4.14
	*R*_p_ (%)	3.08	3.09
	*χ* ^2^	3.48	3.44
Selected bond lengths (Å)			
Mg(1)–O(3)	2.07 (5)	2.08 (4)	
Mg(1)–O(2) (apical)	2 × 2.099 (19)	2 × 2.079 (19)	
Mg(1)–O(7)	2.10 (5)	2.11 (5)	
Mg(1)–O(5)	2.11 (4)	2.13 (4)	
Mg(1)–O(9)	2.27 (5)	2.26 (4)	
Mg(2)–O(1) (apical)	2 × 1.993 (17)	2 × 1.984 (16)	
Mg(2)–O(4)	2.02 (6)	2.01 (5)	
Mg(2)–O(6)	2.04 (5)	2.03 (5)	
Mg(2)–O(10)	2.11 (5)	2.13 (4)	
Mg(2)–O(8)	2.13 (5)	2.13 (5)	
Re(1)–O(5)	1.67 (3)	1.66 (3)	
Re(1)–O(3)	1.97 (4)	1.97 (3)	
Re(1)–O(7)	1.98 (4)	1.97 (4)	
Re(1)–O(9)	2.00 (5)	1.99 (3)	
Re(1)–O(1) (apical)	2 × 2.003 (17)	2 × 2.012 (16)	
Re(2)–O(6)	1.88 (5)	1.88 (4)	
Re(2)–O(10)	1.90 (4)	1.90 (4)	
Re(2)–O(8)	1.92 (4)	1.91 (4)	
Re(2)–O(2) (apical)	2 × 1.930 (19)	2 × 1.947 (19)	
Re(2)–O(4)	2.01 (5)	2.01 (5)	

a Fixed to define origin of unit cell along polar axis.

### 3.5. Magnetic characterisation

**Fig. 9:**
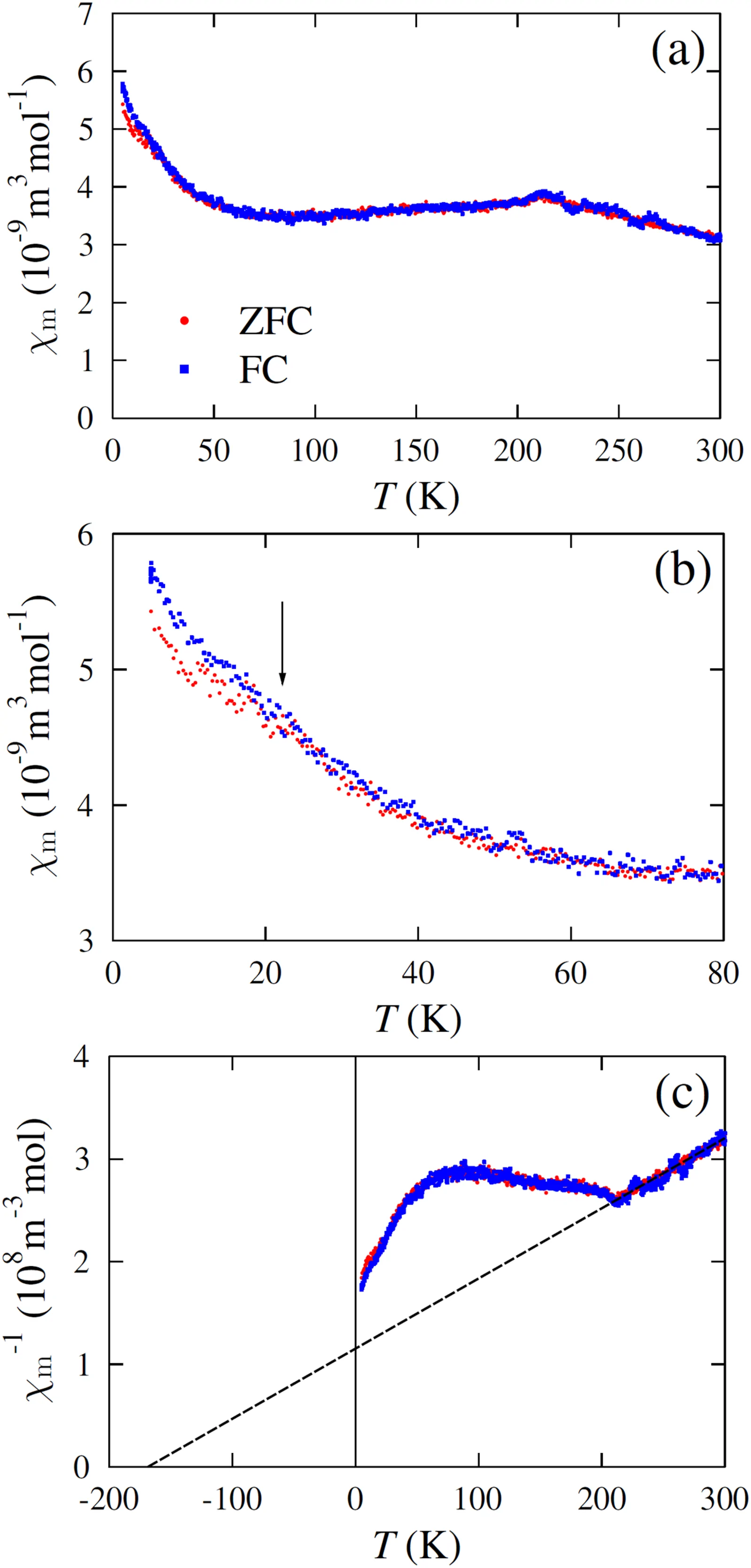
(a) Shows field-cooled (FC, blue) and zero-field-cooled (ZFC, red) magnetic susceptibility data for Pb_2_MgReO_6_ collected on warming in 1000 Oe field; (b) highlights the low temperature region (with arrow marking temperature below which there's a slight divergence of FC and ZFC data) and (c) shows inverse susceptibility *vs.* temperature with a trendline fitted to *T* ≥ 214 K showing Curie–Weiss like behaviour.

The field-cooled (FC) and zero-field cooled (ZFC) magnetic susceptibilities were measured for Pb_2_MgReO_6_ and showed a local maximum in susceptibility at about 214 K (Fig. 9). Above this temperature, the material showed Curie–Weiss-like behaviour (with Weiss temperature *θ* = −169(2) K and estimated paramagnetic moment of 0.965(3)*µ*_B_ per formula unit). This effective paramagnetic moment is comparable to values reported for 5d^1^ double perovskites (*e.g.* 0.67–0.69*µ*_B_ for Ba_2_MgReO_6_,^26^ 0.76(5)*µ*_B_ for Sr_2_ZnReO_6_^65^) and smaller than those reported for Re^5+^ 5d^2^ double perovskites (*e.g.* 1.97*µ*_B_ and 1.93*µ*_B_ for La_2_LiReO_6_ and Ba_2_YReO_6_, respectively^66^). This local maximum in susceptibility seems to coincide with the *R*3̄− to−*Pmc*2_1_ structural phase transition. As shown below, muon-spin relaxation (µSR) experiments at 240 K and 180 K provided no evidence of any magnetic order above or just below this transition. This feature in the magnetic susceptibility may reflect the changes in the Re–O bond lengths and angles at the nuclear phase transition, which in turn change the energy and relative populations of the ground state doublet and excited states, as observed around the tetragonal – monoclinic structural phase transition (∼300 K) in Sr_2_ZnReO_6_.^65^ There is a slight divergence of the FC and ZFC susceptibility data below ∼25 K (Fig. 9b), but given the very small moment, it's hard to confirm the presence of any magnetic order based on these measurements. Measurements of magnetisation *M* with field *H* may help identify magnetic phase transitions, particularly if phases show any field-dependence.

Given the small magnetic moment expected for *A*_2_MgReO_6_-type materials^26^ is it perhaps not surprising that there was no evidence of magnetic Bragg peaks (indicating long-range magnetic order) in low-temperature NPD data for Pb_2_MgReO_6_. Attempts were made to identify magnetic scattering by subtracting 17 K constant wavelength NPD data from the 3.5 K data but no difference was observed. Medium resolution time-of-flight NPD data up to long d-spacing were collected over 5.75 hours at 20 K and 1.5 K and again, subtracting the 20 K data from the 1.5 K to give the difference profile revealed a single small feature at 2.75 Å (close to the 021 peak of the nuclear unit cell) (see SI). It is likely that the tiny feature at 2.75 Å is due to some artefact in the data collection. Magnetic Bragg scattering from possible ordering of magnetic dipoles (assuming the expected Re^6+^ form factor),^67^ would give Bragg peaks at longer d spacings, confirming the lack of evidence for magnetic order from our NPD data, similar to findings by da Cruz Pinha Barbosa *et al.* for related 5d^1^ double perovskites.^8,30^ Reports of long-range magnetic order in related materials Sr_2_MgReO_6_^25^ and Ba_2_MgReO_6_^18,26,68^ prompted us to investigate this possibility using µSR which has recently given important insights into the magnetism of Sr_2_ZnReO_6_.^65^

In a muon-spin relaxation (µSR) experiment, spin-polarised positive muons are implanted in a material, and the muon polarisation is measured as a function of time *via* the asymmetry *A*(*t*).^49^ This polarisation is relaxed by the local magnetic field distribution at the muon sites, so it is sensitive to the local magnetism of a system and its dynamics.

**Fig. 10:**
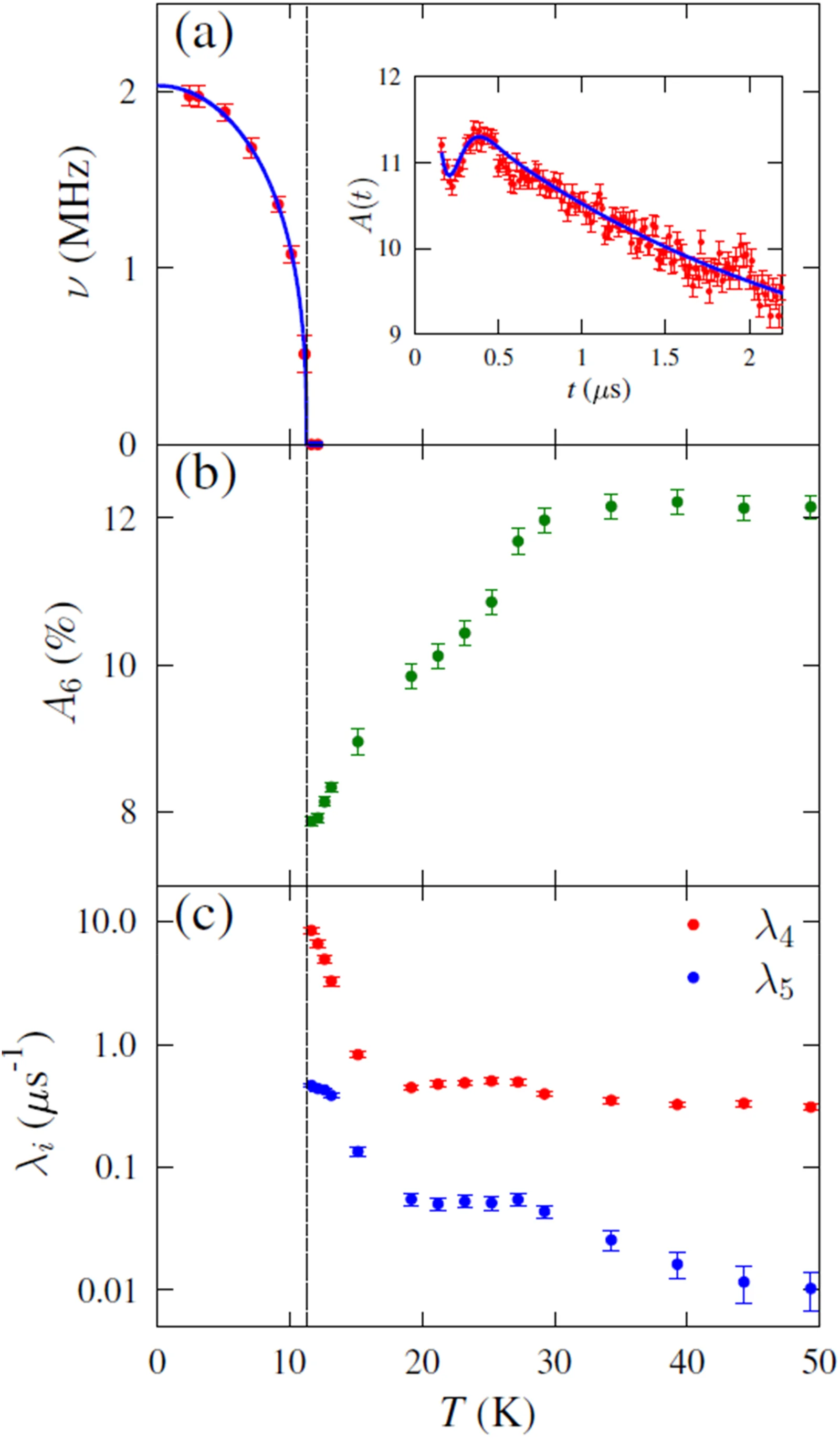
(a) Temperature dependence of the muon precession frequency *ν* with a fit shown in blue. Inset: example ZF µSR spectrum measured at 5 K. (b) Temperature dependence of the time independent contribution to *A*(*t*) in eqn (2). (c) Variation with temperature in the relaxation rates of the components with amplitudes *A*_4_ (red) and *A*_5_ (blue). The transition to LRO at 11.3 K is shown with a dotted line.

Zero-field (ZF) µSR measurements made using the EMU spectrometer revealed oscillations (of frequency *ν*) at low temperatures, indicating the presence of long-range magnetic order in Pb_2_MgReO_6_. The spectra measured for *T* < 11.5 K (Fig. 10a) were fitted with

1 *A*(*t*) = *A*_1_ cos(2π*νt*)e^−*λ*_1_*t*^ + *A*_2_e^−*λ*_2_*t*^ + *A*_3_,

where *A*_1_ = 12(3)%, *A*_2_ = 3.92(2)%, *A*_3_ = 8.03(2)%, *λ*_1_ = 12.4(8) μs^−1^ and *λ*_2_ = 0.45(1) μs^−1^ were found to be temperature independent and were therefore held constant, and *A*_3_ accounts for muons that stop in the sample holder or silver foil packet and so undergo no relaxation.

For muons stopping in regions hosting magnetic order, the oscillating term in eqn (1) describes muon spin components initially oriented perpendicular to the local magnetic field (*B*) direction, causing them to precess at a frequency 
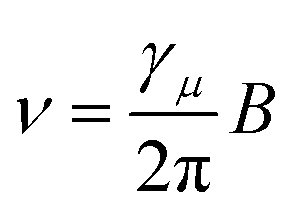
, while the purely relaxing term describes muons spin components initially parallel to the local field direction. The frequency of oscillations, *ν*, displays order parameter-like behaviour and fitting 
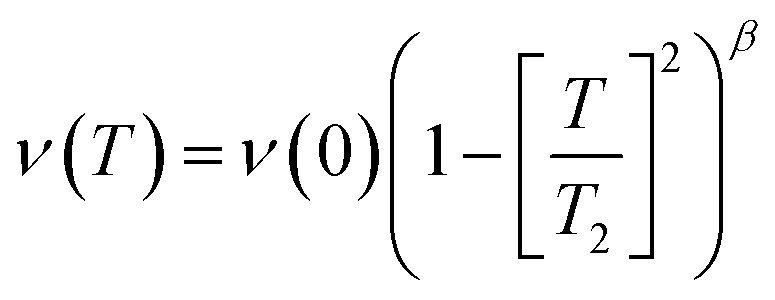
 we obtain *β* = 0.38(4) (Fig. 10a), which is consistent with the value expected for 3D Heisenberg fluctuations. We estimate a transition temperature *T*_2_ = 11.3(1) K and the ground state frequency of *ν*(0) = 2.04(4) MHz, with the latter corresponding to a local field at the muon site of 15.1 mT.

For temperatures 11.5 < *T* < 50 K oscillations are no longer observed, and the measurements suggest significant changes occur in the magnetic behaviour of Pb_2_MgReO_6_. In this region a two-exponential model,

2 *A*(*t*) = *A*_4_*e*^−*λ*_4_*t*^ + *A*_5_*e*^−*λ*_5_*t*^ + *A*_6_,

captures the behaviour of the spectra, with *A*_4_ = 3.3(1) % and *A*_5_ = 4.60(4)% held constant. We observe a large (*λ*_4_) and a small (*λ*_5_) relaxation rate that undergo similar variations with temperature but differ in size by an order of magnitude. Immediately above *T*_2_ = 11.3(1) K, both relaxation rates decrease rapidly, then remain roughly constant in the region 20 ≲ *T* ≲ 30 K, before gradually decreasing at higher temperatures. This behaviour can be explained by the presence of short-ranged magnetically ordered regions above *T*_2_, that are dynamically fluctuating. Such a picture would cause the muon-spin components perpendicular to the internal field to be rapidly relaxed by a combination of the static magnetism (that varies with position), and dynamic magnetic fluctuations, giving rise to the larger relaxation rate *λ*_4_. The muon spin components parallel to the internal field can only be relaxed by the dynamic magnetic fluctuations and would then give rise to the smaller relaxation rate *λ*_5_.

The constant contribution to the asymmetry, *A*_6_ (which reflects non-magnetic regions of the material that do not relax the muon spins), displays changes in behaviour at similar temperatures to the relaxation rates. Amplitude *A*_6_ increases from 8% at *T*_2_ to 12% at 30 K, with a clear change in behaviour at 20 K, and is approximately constant for *T* > 30 K. The temperature *T*_1_ ≈ 30 K (Fig. 10b), therefore marks the upper-bound of a crossover region in magnetic behaviour in Pb_2_MgReO_6_. This implies that the short-range ordered regions shrink in volume with increasing temperature in the region 11 ≲ *T* ≲ 30 K.

Weak transverse field muon measurements were also performed (see SI), whose results are consistent with the zero field results described here. In particular, these measurements show additional evidence for an increase in the non-magnetic volume fraction of the material above 11 K (see Fig. S17).

To investigate possible magnetic structures for Pb_2_MgReO_6_, muon site calculations were performed (see SI for details). We identified a low energy cluster of magnetically inequivalent muon sites with similar internal field, explaining the broad, single-frequency response seen in data below *T*_2_ = 11.3 K. The dipole field at these muon sites was calculated for several candidate magnetic structures, with the best fit to the experimental data obtained for an antiferromagnetic order of Re^6+^ spins (see SI). The local field distribution at the muon sites for this AFM structure has a single broad peak of width 7.7 mT (1.0 MHz). Matching the average local field of this distribution to the measured frequency *ν*(*T* = 0) gives a moment of *μ*_Re_ = 0.16(6)*μ*_B_, similar to the value seen in other *A*_2_MgReO_6_ double perovskites.^18,26,30^ A number of other antiferromagnetic and ferromagnetic ordering patterns for the Re^6+^ spins were also considered but these resulted in more than one distinct peak in the field distribution at the muon sites. Measurements of magnetisation with field would be of interest to determine whether any of these phases have any ferromagnetic components, and may be useful for future work to distinguish between possible magnetic phases.

Finally, we also investigated temperatures close to the structural phase transition at 210 K. In both ZF and wTF measurements at 180 K and 240 K we observe little variation in the spectra either side of the transition, despite a small magnetic response being observed in this region in the susceptibility. This suggests that the structural transition occurring in this temperature regime gives rise to a change in the average response of the magnetization to an applied magnetic field, *e.g. via* a change in the separation of crystal field levels of the magnetic ions, leading to differences in the occupation of the levels. However, at these temperatures, the moments will likely be rapidly fluctuating on the muon timescale, such that magnetic fluctuations are motionally narrowed from the muon spectrum. It is therefore not necessarily significant that a response is not observed by the muon around 210 K.

## 4. Discussion

The structural behaviour of *A*_2_*BB*′O_6_ double perovskites, and the resulting site symmetry of magnetic *B*′ cations, play a key role in determining their properties and magnetic ground states. The orthorhombic ground state described here for Pb_2_MgReO_6_ is reached *via* a first-order phase transition from an *R*3̄ (*a*^−^*a*^−^*a*^−^) high temperature phase on cooling through ∼200–210 K. There is only a relatively small entropy change (∼0.2 J mol^−1^ K^−1^, Fig. 2) associated with this phase transition, similar to the *Fm*3̄*m* – *R*3̄ tilt transition at ∼370 K in double perovskite Pb_2_CoTeO_6_^40^ but noticeably smaller than that observed for comparable phase transitions (*e.g.* in Pb_2_MnWO_6_, Pb_2_CdWO_6_, Pb_2_MnReO_6_, Pb_2_MgTeO_6_).^37,39,64,69^ The low entropy change in related materials has been ascribed to residual disorder (*e.g.* in Pb^2+^ or oxide sites) in the low-temperature phase, reflected by high atomic displacement parameters (ADPs). The Pb^2+^ sites in Pb_2_MgReO_6_ have higher ADPs in the high temperature *R*3̄ phase (Table 1), but all sites have fairly low ADPs (reflecting high order) in the refined 3.5 K and 17 K models (Table 2). As noted above, there is a very small local maximum in magnetic susceptibility (the averaged response of the magnetisation to the applied field) associated with this structural phase transition (Fig. 9a).

The large orthorhombic unit cell (with parameters *a* = 2*a*_p_, 
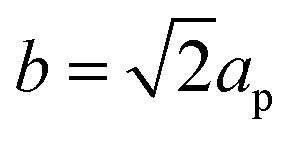
 and 
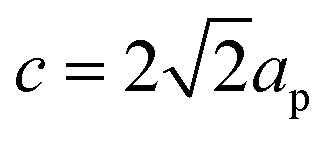
, relative to the ideal simple *Pm*3̄*m* perovskite) results from in-plane antipolar displacements of *A* site cations. Such structural models, of *Pnma* or *Pmc*2_1_ symmetry, are almost exclusively observed for *A* = Pb^2+^ phases^35,39,64,70^ (an exception is (Ca,Sr)ZrO_3_^71^), highlighting the preference of the 6s^2^ Pb^2+^ cation for lower symmetry coordination environments.^34^ Both *Pnma* and *Pmc*2_1_ models involve displacements of both Pb^2+^ sites in the unit cell in antiparallel directions along the short in-plane axis (Fig. 8). Displacements are of equal magnitude for the two sites for the antipolar *Pnma* model. However, the polar *Pmc*2_1_ model, described here for Pb_2_MgReO_6_ at low temperatures, allows displacements of different magnitudes for the two sites, giving rise to an uncompensated polarisation along the *c* axis (as discussed in the SI), also observed for Pb_2_MnWO_6_.^35^

Experimental and theoretical work on *A*_2_MgReO_6_ materials as candidates for quadrupolar order has highlighted the coupling between magnetic order and symmetry lowering structural distortions. *A* hallmark of the quadrupolar order is equatorial stretching/contraction of ReO_6_ octahedra along [110] directions of the *Fm*3̄*m* unit cell (described by X_2_^+^ irrep^29,30^) which, alone, would lower the crystal symmetry to *P*4_2_/*mnm*.^28,30,72^ There is no evidence of these distortions in the *Pnma* or *Pmc*2_1_ models described here for Pb_2_MgReO_6_. Any reduction of Re ions caused by oxide vacancies (see Section 3.2) is likely to disrupt any tendencies towards quadrupolar order.

Although no signatures of low temperature phase transitions are observed in heat capacity data (Fig. 2), analysis of our µSR results (Fig. 10 and Fig. S6) suggests that regions of short-range magnetic order exist in Pb_2_MgReO_6_ for 11.3 ≲ *T* ≲ 30 K, with the size of the ordered regions increasing on cooling until below *T*_2_ = 11.3 K, long-range AFM ordering of Re spins occurs. A similar situation, where muons are sensitive to transitions that are not resolved *via* heat capacity, has been observed in many systems.^49^ For temperatures above *T*_1_ = 30 K, the non-magnetic fraction of the sample is constant on the muon timescale. The lack of response in the heat capacity to the magnetic ordering is consistent with the occurrence of short-range order at higher temperatures. If such short-range order occurs, the entropy change that occurs when the system undergoes a transition to long-range order at lower temperature will be much reduced compared to a state of affairs where the transition is between disorder and long-range order. As a result of this limited entropy change, the response of heat capacity is lessened.

The paramagnetic moment determined here for Pb_2_MgReO_6_ (0.965(3)*µ*_B_) is comparable to those reported for tetragonal 5d^1^ double perovskites Ba_2_CdReO_6_ and Sr_2_LiOsO_6_. It is noticeably reduced from the spin-only value (1.73*µ*_B_) consistent with some quenching of the orbital moment due to the Re site symmetry^30^ but larger than that reported for higher symmetry 5d^1^ double perovskites (Re sites of the following symmetries: 3̄ in the *R*3̄ phase; *m* in the *Pnma* or *Pmc*2_1_ orthorhombic phases; *mmm* in the *P*4_2_/*mnm* quadrupolar phase and *m*3̄*m* in the parent *Fm*3̄*m* phase). We note it is also smaller than that typically reported for 5d^2^ Re^5+^ double perovskites.^66^

The slight divergence of FC and ZFC magnetic susceptibility data below *T*_1_ = 30 K (Fig. 8), the paramagnetic moment (0.965(3)*µ*_B_), and the suggestion of short-range magnetically ordered regions for 11.3 ≲ *T* ≲ 30 K for Pb_2_MgReO_6_ are similar to Sr_2_LiOsO_6_ and the report of its partially frozen magnetic state below 30 K.^8^ This 5d^1^ double perovskite adopts a tetragonal structure (of *I*4/*m* symmetry structure) even at room temperature, in order to optimise bonding around the Sr^2+^ ions on the perovskite *A* sites. The distortion at 200–210 K described here for Pb_2_MgReO_6_ also arises due to the bonding requirements of the *A* cation, in this case the 6s^2^ Pb^2+^ ion: the lower symmetry orthorhombic structure is consistent with the second-order Jahn–Teller (SOJT) distortion of this ion. Although these structural distortions occur at much higher temperatures than magnetic and orbital ordering effects, the resulting changes in symmetry of the Re^6+^/Os^5+^ sites are key in determining the magnetic behaviour at low temperatures: da Cruz Pina Barbosa *et al.* highlight the importance of the orbital order of the *P*4_2_/*mnm* phase to realise the heavily canted AFM order reported for Ba_2_MgReO_6_.^8,30^ The electronic driving force for more complex orthorhombic distortions in Pb_2_MgReO_6_ prevents this orbital ordering (in addition to any effects from reduction of the Re ions) and hence a collinear AFM magnetic order is thought to occur below *T*_2_ = 11.3 K. For the Kramers Re^6+^ ion, symmetries below cubic split the Γ_8_ state into two doublets, and the further symmetry lowering (*e.g.* ..*m* in orthorhombic *Pnma* and *Pmc*2_1_ models; 3̄ in the *R*3̄ phase) does not split the ground state doublet further. However, the structural phase transition at 200–210 K may change the energy gap between the ground state and excited states, changing their relative populations of these states and their contributions to the magnetic behaviour.^65^

## 5. Conclusions

A diversity of magnetic ground states has been reported for double perovskites containing 5d^1^ magnetic cations which have been shown to be sensitive to the balance between spin–orbit coupling, cation site symmetry and exchange interactions.^9,10^ The role of tolerance factor and octahedral rotations in tuning cation site symmetry and magnetic behaviour has been explored^8,30^ and this current study on Pb_2_MgReO_6_ demonstrates a similar role of the SOJT Pb^2+^ ions on the perovskite *A* sites. This gives rise to a structural phase transition at 200–210 K, giving a large orthorhombic unit cell and structure similar to those reported for Pb_2_MnWO_6_^35^ and Pb_2_MgWO_6_.^38^ This lowers the symmetry of Re^6+^ sites such that it is incompatible with *P*4_2_/*mnm* orbital ordering, hence it prevents the heavily canted FM order associated with Ba_2_MgReO_6_ and Ba_2_NaOsO_6_ phases.^9,26,68^ The crystal structure is dominated by Pb^2+^ displacements and the relationship between the reported polar *Pmc*2_1_^35^ and antipolar *Pnma*^37,38^ phases is understood in terms of distortion modes and relative displacements of the two Pb^2+^ sites.

## Conflicts of interest

There are no conflicts to declare.

## Supplementary Material

TC-014-D6TC01983H-s001

## Data Availability

The data supporting this article have been included as part of the supplementary information (SI) as appropriate. Supplementary information is available. See DOI: https://doi.org/10.1039/d6tc01983h. Neutron powder diffraction (raw, unfocused) (and constant wavelength data) and muon spin relaxation data are available at DOIs https://doi.org/10.5286/ISIS.E.RB2210115, https://doi.org/10.5291/ILL-DATA.5-31-3049 and https://doi.org/10.5286/ISIS.E.RB2220119, respectively. Crystallographic data for Pb_2_MgReO_6_ will be deposited at the ICSD on acceptance of the manuscript for publication.
