# Supplementary material for: Structural distortions and consequences for magnetism in the 5d1 double perovskite Pb2MgReO6
Source: J Mater Chem C Mater. 2026 Jul 28;14(35):16403–17. doi: 10.1039/d6tc01983h (PMC13584819; doi:10.1039/d6tc01983h)
Supplement: Supplementary file 1 [file TC-014-D6TC01983H-s001.pdf]

Supplementary Information (SI) for Journal of Materials Chemistry C.
This journal is © The Royal Society of Chemistry 2026

Structural distortions and consequences for magnetism in the 5d[1] double perovskite Pb_2MgReO_6

Hai L. Feng,[1,2] Martha Greenblatt,[1] Nathan P. Bentley,[3] Tom Lancaster,[3] Adrian D. Hillier,[4] Budhika Mendis,[3] Rebecca Rae,[5] Fabio Orlandi,[4] Emma Suard,[6] Thomas J. Hicken,[7] Emma E. McCabe[3]*

[1]Department of Chemistry and Chemical Biology, Rutgers, the State University of New Jersey, Piscataway, New Jersey 08854, United States
[2]Beijing National Laboratory for Condensed Matter Physics and Institute of Physics, Chinese Academy of Sciences, Beijing, 100190, China
[3]Department of Physics, Durham University, Durham DH1 3LE, U.K.
[4]ISIS Neutron and Muon facility, STFC, Rutherford Appleton Laboratory, Harwell Campus, Didcot, Oxfordshire OX11 0QX, U.K.
[5]School of Physics and Astronomy, University of Edinburgh, Edinburgh, EH9 3JZ, U.K.
[6]Institut Laue-Langevin, 71 Avenue des Martyrs, 38042 Grenoble, France
[7]PSI Center for Neutron and Muon Sciences, 5232 Villigen PSI, Switzerland

Supporting information:

Structural refinements for Pb_2MgReO_6 using 250 K NPD data

Initial refinements were carried out using a cubic model of $Fm\bar{3}m$ symmetry allowing ordering of Mg^{2+} and Re^{6+} ions over the *B* sites but with no tilts of BO_6 octahedra, as reported for Ba_2MgReO_6. Refinement profiles and structural details are given in Table SI1 and Figure SI1.

Table SI1 Details from Rietveld refinement using 250 K NPD data for Pb_2MgReO_6 using a cubic model of $Fm\bar{3}m$ symmetry; *a* = *b* = c = 9.64(2) Å, volume = 505.14(12) Å^3.

**Atom**	**Wyckoff site**	**x**	**y**	**z**	**U_{iso} × 100 (Å^3)***
Pb	8*c*	0.25	0.25	0.25	1.46(12)
Mg	4*b*	0.5	0.5	0.5	1.46(12)
Re	4*a*	0	0	0	1.46(12)
O	24*4*	0.2356(3)	0	0	1.46(12)

* Values constrained to equal a single global value for this atomic displacement parameter

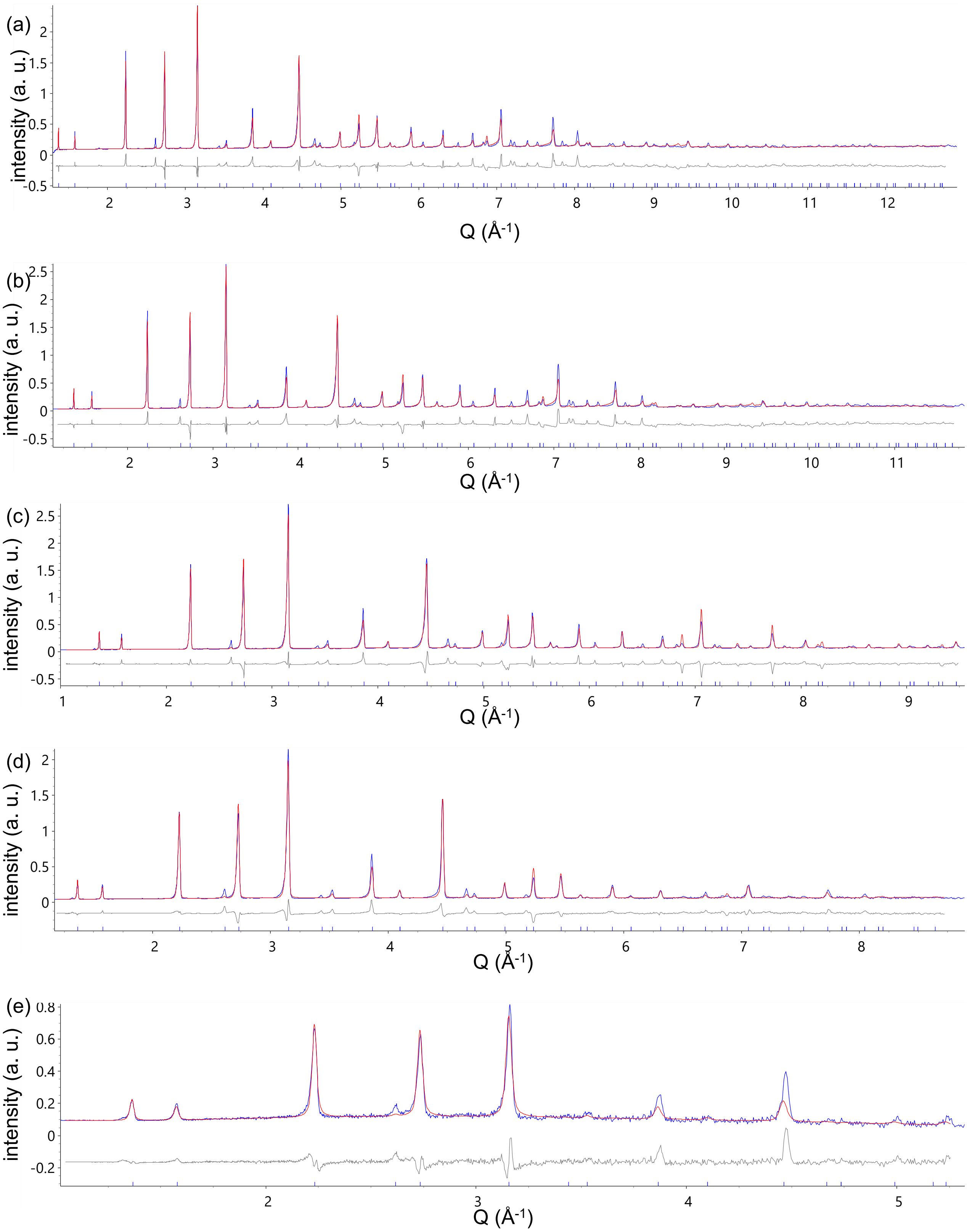

Figure SI1 Rietveld refinement profiles for 250 K NPD data for Pb_2MgReO_6 using a model of $Fm\bar{3}m$ symmetry showing (a) bank 5 (153°), (b) bank 4 (122°), (c) bank 3 (90°), (d) bank 2 (58°) and (e) bank 1 (27°) banks of data. Observed, calculated and difference profiles are shown in blue, red and grey, respectively; R_{wp} = 11.14%, R_p = 8.61% and χ^2 = 19.04.

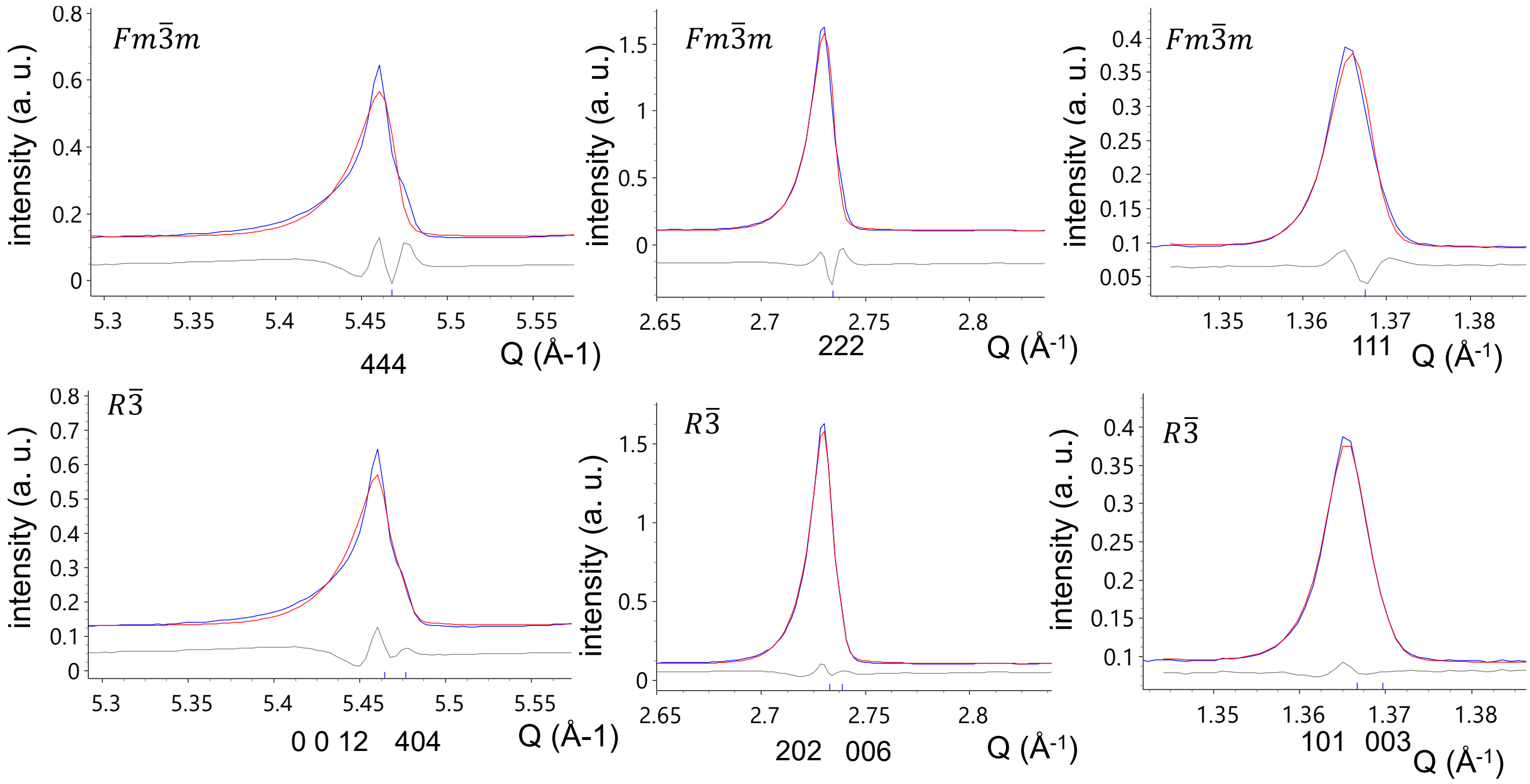

Figure SI2 Enlarged view of key diffraction peaks in high resolution data bank showing comparison of fit from Pawley refinements using (top) $Fm\bar{3}m$ unit cell and (bottom) $R\bar{3}$ unit cell. Observed, calculated and difference profiles are shown in blue, red and grey, respectively. Peak positions are indicated by tick marks and *hkl* indices for these reflections are given below.

Structure refinements using low temperature nuclear structure of Pb_2MgReO_6

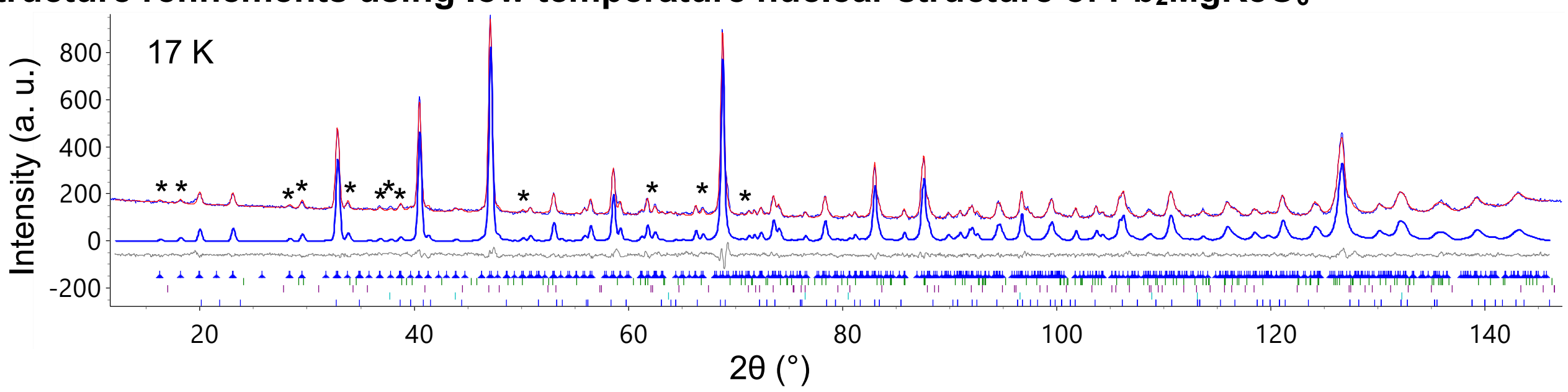

Figure SI3 Rietveld refinement profiles showing fits to $Pmc2_1$ models for Pb_2MgReO_6 at 17 K; observed, calculated and difference profiles are shown in blue, red and grey, respectively and top (blue), upper (green), lower (purple), lower (cyan) ticks show peak positions of main phase (98(9)% by weight), PbO_2 (1.2(6)% by weight), PbO (0(9)% by weight) and MgO (1.1(9)% by weight), and bottom ticks (blue) show peaks predicted by the high symmetry $R\bar{3}$ model. Significant peaks not indexed by this $R\bar{3}$ model are highlighted with asterisks.

Symmetry analysis for low temperature nuclear structure of Pb_2MgReO_6

Indexing the diffraction peaks for the low temperature (T < 200 K) phase indicates that a large, primitive orthorhombic cell is needed to describe this phase, with approximate unit cell parameters 11.3 Å × 8.0 Å × 5.6 Å (with transformation matrix (1 1 0) (0 0 1) (½ -½ 0) relative to the parent $Fm\bar{3}m$ model). Symmetry analysis using ISODISTORT[1, 2] suggests this is consistent with distortions described by the Σ_2 irrep (k = ½ ½ 0), giving models of $Pnma$, $Pmma$ or $Pmc2_1$ symmetries for different directions of this high-dimensional order parameter. A model of $P1$ symmetry with 11.3 Å × 8.0 Å × 5.6 Å unit cell was constructed using ISODISTORT,[1, 2] to allow us to consider possible low-symmetry models in terms of the parent $Fm\bar{3}m$ model with additional symmetry-adapted distortion modes applied. The amplitudes of these modes can be refined (in symmetry-adapted groups,[3, 4] or

individually[5, 6], often using simulated annealing, (referred to as "mode inclusion analysis" in the subsequent text) to find the distortion modes that give the greatest improvement in fit.

Mode inclusion analysis of our NPD data (17 K and 3.5 K data collected on D2B (ILL)), Figure SI4, indicate that refining the amplitudes of Σ_2 modes to describe in-plane antipolar displacement of Pb^{2+} cations gives the biggest improvement in fit.

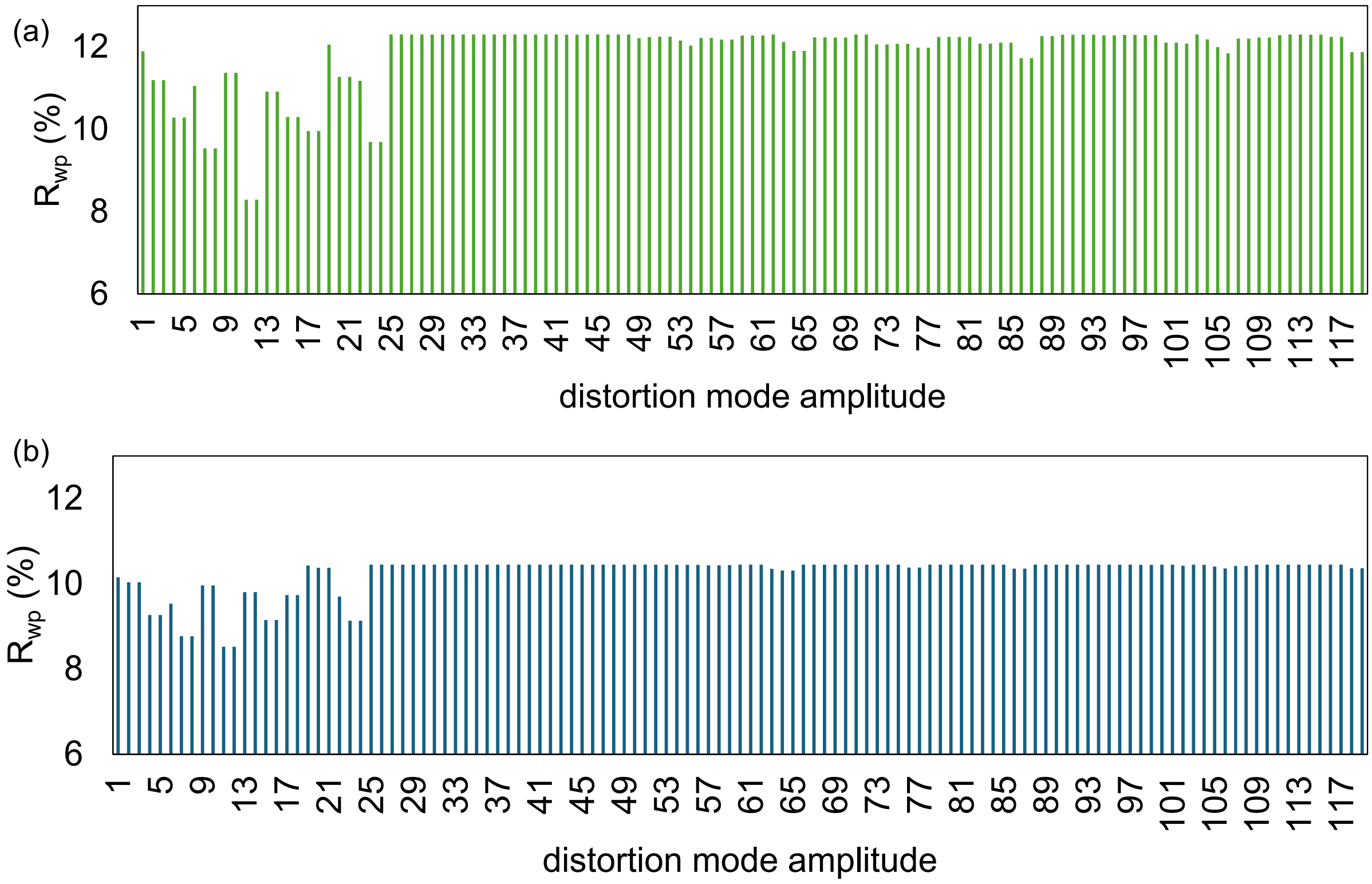

Figure SI4 Results from a first round of mode inclusion analysis for (a) 17 K NPD data and (b) 3.5 K NPD data collected for Pb_2MgReO_6 showing that the fit improves significantly if the amplitudes of modes 11 or 12 (Pb^{2+} Σ_2 modes) are allowed to refine.

In our $P1$ model, there are two Σ_2 modes to describe Pb^{2+} displacements. In the $Pnma$ model, both modes would be constrained to be of equal amplitude and would give in-plane antipolar displacements of all Pb^{2+} sites. The $Pmma$ model would correspond to only one of these modes having non-zero amplitude and would allow only half the Pb^{2+} sites to undergo this in-plane antipolar displacement. The more complex $Pmc2_1$ model would require both modes to have non-zero amplitudes, but of different magnitudes, hence giving an overall in-plane polarisation.

A surface plot showing the fit to the observed data (measured by R_{wp}) as a function of the two Pb^{2+} Σ_2 mode amplitudes (Figure 6 of the main text) indicates that at 17 K, the best fit is obtained with non-zero amplitudes of both these modes, ruling out the model of $Pmma$ symmetry. However, rather than observing a strict 1:1 correlation between the amplitudes of the two modes (e.g. $\Sigma_{2\_Pb(1)} = \Sigma_{2\_Pb(2)}$ as expected for a $Pnma$ model), the mode amplitudes are correlated but are not necessarily of equal magnitudes.

On cooling further to 3.5 K (Figure 6 of the main text), again both Pb^{2+} Σ_2 mode amplitudes are clearly non-zero in models that give the best fits, but it is also clear that the best fits are found for models with a clear difference in amplitudes for the two modes, indicating greater deviation from the $Pnma$ model towards the polar $Pmc2_1$ model.

Preliminary refinements were carried out using $Pnma$ and $Pmc2_1$ models to fit 17 K and 3.5 K data and both gave good fits (Table SI2):

Table SI2 Summary of Rietveld refinement fitting statistics for 3.5 K and 17 K NPD data for $Pnma$ and $Pmc2_1$ models for Pb_2MgReO_6.

**Model**	**3.5 K**	**17 K**
$Pnma$	R_{wp} = 5.18% (38 parameters)	R_{wp} = 5.21% (38 parameters)
$Pmc2_1$	R_{wp} = 4.43% (56 parameters)	R_{wp} = 4.39% (56 parameters)

The improvement in fit for the polar $Pmc2_1$ model suggests this extra complexity is justified. To test this, mode inclusion analysis was carried out starting from the $Pnma$ model to compare the improvement in fit from refining the amplitudes of symmetry-adapted groups of modes that would lower the symmetry. The possible symmetry-lowering distortions are summarised in Table SI3.

Table SI3 Summary of symmetry-lowering distortions from the $Pnma$ model to be compared in mode inclusion analysis.

**Mode**	**Resulting symmetry and transformation matrix**	**Description of symmetry-lowering distortion**
Γ_2^+	$P2_1/c$, (1,0,0),(0,1,0),(0,0,1)	rotation of BO_6 octahedra about an in-plane axis
Γ_3^+	$P2_1/c$, (0,1,0),(0,0,1),(1,-1,0)	rotation of BO_6 octahedra about an in-plane axis, but some distortion of octahedra
Γ_4^+	$P2_1/m$, (0,-1,0),(1,0,0),(0,0,1)	out-of-phase rotation of BO_6 octahedra about an out-of-plane axis
Γ_1^-	$P2_12_12_1$, (0,0,1),(1,0,0),(0,1,0)	rotation of BO_6 octahedra about an in-plane axis, but some distortion of octahedra
Γ_2^-	$Pmn2_1$, (1,0,0),(0,0,1),(0,-1,0)	polarisation along the shorter in-plane axis
Γ_3^-	$Pmc2_1$, (1,0,0),(0,1,0),(0,0,1)	polarisation along the longer in-plane axis
Γ_4^-	$Pna2_1$, (0,0,1),(0,1,0),(-1,0,0)	polarisation along the out-of-plane axis

At both temperatures, the greatest improvement in fit was observed on allowing the amplitudes of Γ_3^- modes to refine (Figure SI5), consistent with a symmetry lowering to polar $Pmc2_1$, with polarisation along the longer in-plane axis.

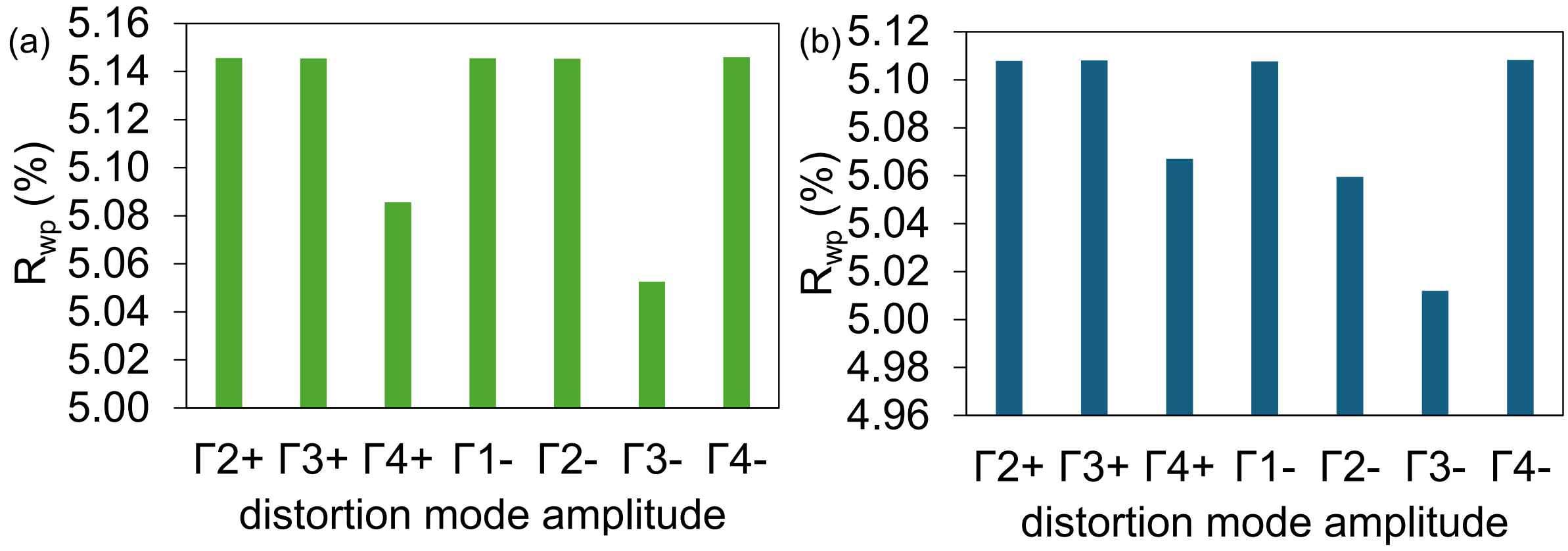

Figure SI5 Mode inclusion analysis for (a) 17 K NPD data and (b) 3.5 K NPD data collected for Pb_2MgReO_6 showing that the fit improves slightly if the symmetry is lowered from $Pnma$ by refining the amplitudes of Γ_3^- modes, lowering the symmetry to $Pmc2_1$.

The improvement in fit is small but seems significant and justified compared with the smaller improvements in fit observed for alternative lower-symmetry models. The $Pmc2_1$ models refined to fit the 3.5 K and 17 K data are very similar and it is hard to identify significant differences in specific atom sites once final refinements with atomic displacement parameters have been carried out. Compared with the high symmetry $Fm\bar{3}m$ model for *B*-site ordered double perovskites, the dominant distortions are those of Γ_5^+ symmetry (antipolar displacements along the out-of-plane *a* axis), Γ_4^- symmetry (polar displacements along the long in-plane *c* axis), Σ_2 displacements described above,

X_4^+ displacements of equatorial O sites along the long in-plane c axis, and X_5^- antipolar displacements (predominantly of Pb^{2+} sites along the long in-plane c axis. The result of the Σ_2 and X_5^- distortions on the Pb^{2+} sites is illustrated in Figure 8 of the main text.

Muon site calculations and possible low-temperature magnetic structure of Pb_2MgReO_6

Muon site calculations performed on Pb_2MgReO_6 identified a cluster of 8 candidate muon sites (Table SI4) that are closely spaced in energy and 0.7 eV lower in energy than any others identified. It is reasonable to suggest that these low-energy sites are the only sites that could be realised in Pb_2MgReO_6. These sites occupy the 4c Wykoff position in the unit cell, with the local environment dominated by an oxygen site located 1.0-1.5 Å from the muon. The sites can be categorised into two groups, A and B, based on their approximate locations (within 0.3 Å of each other and coupled to the same oxygen, Table SI4). Group A comprises sites 1 and 3, whilst Group B contains sites 4 and 7. Group A sites are close to the apical O(2) site near the Pb(2) chains, whilst Group B sites are close to the equatorial edge of Mg(1)O_6 octahedra in between Pb(2) sites (Figure 8).

Table SI4 Approximate locations for the two groups of low-energy candidate muon sites in Pb_2MgReO_6, with the average energy relative to the lowest-energy muon site.

Group	Site	Coordinates	Average energy (meV)
A	1	(0.267, 0.340, 0.773)	0
	2	(0.339, 0.746, 0.235)	78
A	3	(0.260, 0.337, 0.771)	83
B	4	(0.499, 0.895, 0.892)	84
	5	(0.235, 0.842, 0.024)	96
	6	(0.003, 0.922, 0.803)	104
B	7	(0.502, 0.850, 0.898)	107
	8	(0.135, 0.236, 0.208)	137

The dipole field at these muon sites was then calculated for a range of magnetic structures, making use of the MuESR program.[7] The number of magnetically inequivalent muon sites identified in Pb_2MgReO_6 leads to a spread in the local field values across the muon sites. This distribution results in some static magnetic disorder experienced by the implanted muons and consequently gives a rapid relaxation of the oscillations. The range of muon sites, therefore explains the broad, single-frequency response seen in data below $T_2 = 11.3$ K. Similar behaviour is seen for another 5d^1 double perovskite Sr_2ZnReO_6 for which a large number of low energy symmetry-inequivalent muon sites were also observed.[8]

The magnetic models that give the best fit to the μSR data have Re^{6+} moments (~0.16(6) μ_B) oriented along [100] direction to give AFM magnetic phases. There are several possible magnetic symmetries consistent with this analysis but the small size of the ordered Re^{6+} moments prevented us detecting this magnetic order from NPD data. Figure SI7(c) below shows the difference in NPD data collected at 1.4 K and 20 K which give no evidence for long range dipolar magnetic order.

Other magnetic ordering patterns for the Re^{6+} spins were also considered, but these resulted in more than one distinct peak in the field distribution at the muon sites. Therefore, if all the muon sites are occupied, these models would give multiple frequences in the μSR spectra (as observed for Ba_2NaOsO_6 for example[9]). However, since each candidate muon site has a capture cross section associated with the muon implantation process that is not possible to compute, it is possible that only a subset of the identified muon sites are occupied. For example, if only group B sites have a sizeable capture cross section, then FM ordering of Re^{6+} spins along [100] gives a single peak of width 2.2 mT (0.3 MHz) in the field distribution, resulting in $\mu_{Re} = 0.5(1)\,\mu_B$. As a result, it is not possible to confirm a specific AFM ordering pattern.

Weak transverse-field (wTF) μSR measurements on Pb_2MgReO_6

We also performed wTF muon measurements between 5 and 50 K to probe changes in the magnetic volume fractions of Pb_2MgReO_6. Fitting the spectra to

$$A(t) = A_7 \cos(\gamma_\mu B_{\mathrm{wTF}} t)\, e^{-\lambda_7 t} \tag{3}$$

with $B_{\mathrm{wTF}} = 15(1)$ mT, allows A_7 to describe the variation of the non-magnetic volume fraction with temperature (Figure SI6). Changes in A_7 with temperature are like the constant term in the ZF measurements, which is to be expected as both describe the response of muons stopping in non-magnetic regions. Relaxation rate λ_7 has a peak in temperature near to T_2, reflecting the increase in magnetic fluctuations occurring in the vicinity of a phase transition to a long-range ordered phase.

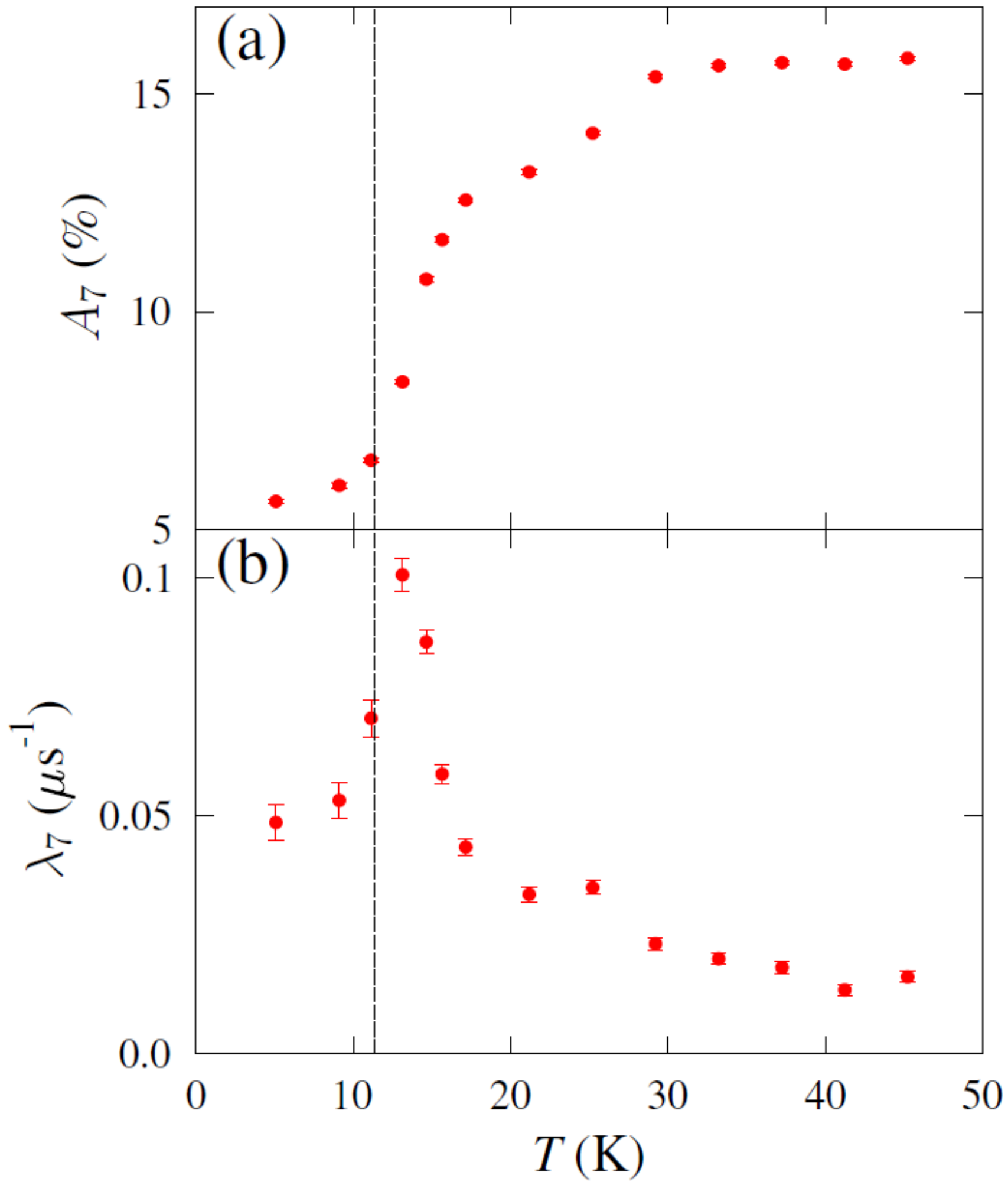

Figure SI6 wTF μSR measurements showing the variation of the (a) amplitude and (b) relaxation rate corresponding to muons implanting in non-magnetic regions of the sample such that they precess about the applied transverse field. The transition to LRO from the ZR μSR analysis is shown by the dotted line.

Lack of magnetic Bragg peaks in low temperature NPD data for Pb_2MgReO_6

Further time-of-flight NPD data were collected in medium resolution mode on the WISH diffractometer at 1.4 K and 20 K. Subtracting the 20 K data from the 1.4 K data did not reveal any additional scattering (that could arise from magnetic order) in the 1.4 K data set, except for a tiny feature at 2.75 Å (Q = 2.285 $Å^{-1}$) in the bank 3 set of data (average 2θ=90°). Rietveld refinements using the nuclear model to the data are shown in Figure SI7 showing almost identical fits. The difference pattern for the same detector bank (from subtracting the 20 K data from the 1.4 K data) are shown in Figure SI7(c) emphasising the lack of magnetic scattering, and only the tiny feature at

2.75 Å. The lack of any Bragg peaks at longer d spacing rules out most models with dipolar magnetic order.

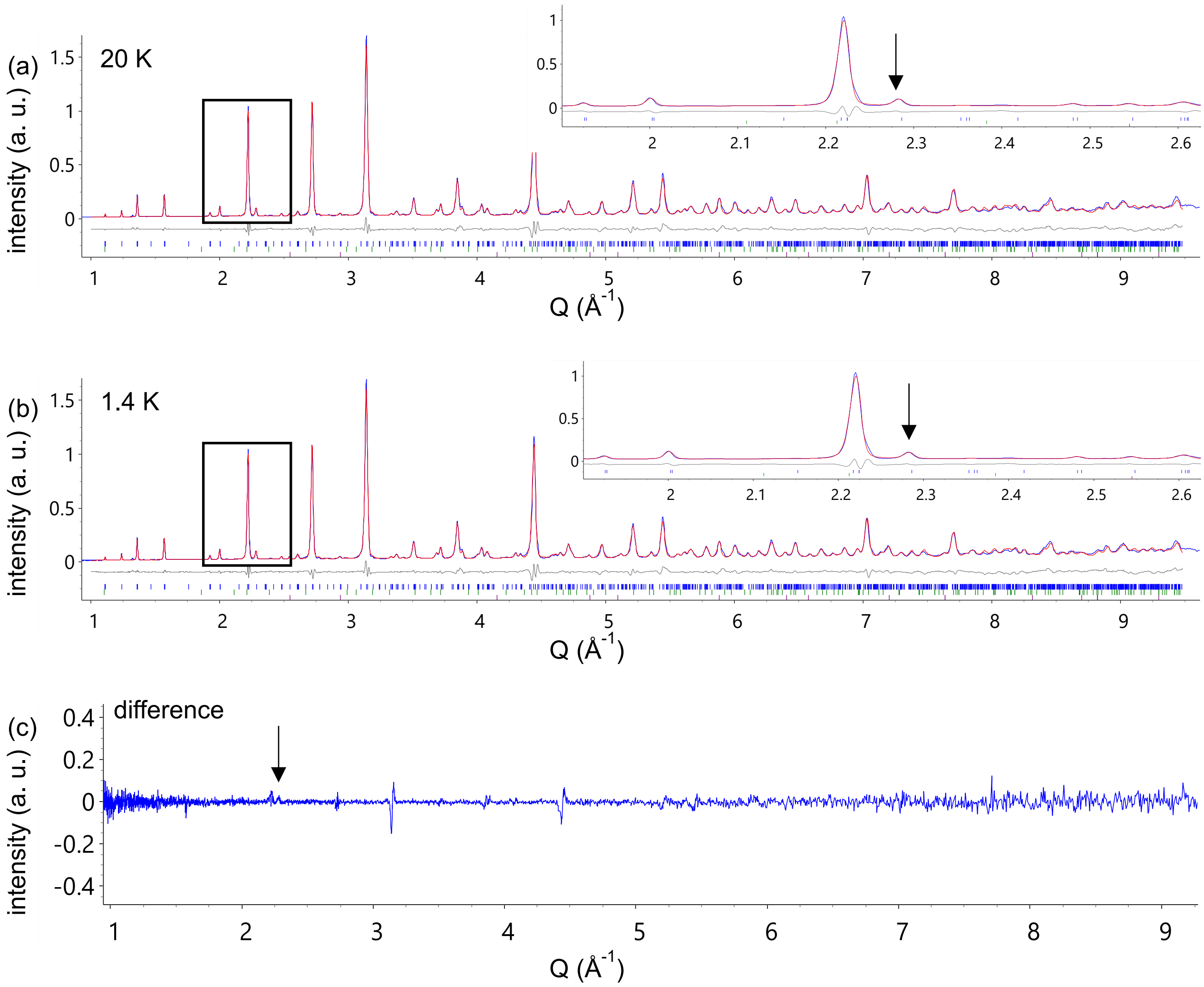

Figure SI7 Rietveld refinement profiles using bank 3 (90°) for Pb_2MgReO_6 at (a) 20 K and (b) 1.4 K showing observed, calculated and difference profiles are shown in blue, red and grey, respectively. Top (blue), upper (green), lower (purple), lower (cyan) ticks show peak positions of main phase (98(9)% by weight), PbO_2 (1.2(6)% by weight), PbO (0(9)% by weight) and MgO (1.1(9)% by weight). Panel (c) shows the difference profile which shows spikes in the positions of the most intense nuclear Bragg peaks (presumably due to the slight change in unit cell parameters between 1.4 – 20 K) and the small feature at 2.75 Å. This region is highlighted in the enlarged insets to panels (a) and (b).

References

(1) Stokes, H. T.; Hatch, D. M.; Campbell, B. J. *ISODISTORT, ISOTROPY Software Suite*. iso.byu.edu (accessed.

(2) Stokes, H. T.; Hatch, D. M.; Campbell, B. J.; Tanner, D. E. ISODISPLACE: a web-based tool for exploring structural distortions. *J. Appl. Cryst.* **2006**, *39* (4), 607-614. DOI: https://doi.org/10.1107/S0021889806014075.

(3) Giddings, A. T.; Scott, E. A. S.; Stennett, M. C.; Apperley, D. C.; Greaves, C.; Hyatt, N. C.; McCabe, E. E. Symmetry and the Role of the Anion Sublattice in Aurivillius Oxyfluoride Bi2TiO4F2. *Inorg. Chem.* **2021**, *60* (18), 14105-14115. DOI: 10.1021/acs.inorgchem.1c01933.

(4) Scott, E. A. S.; Mitoudi Vagourdi, E.; Johnsson, M.; Cascos, V.; John, F.; Pickup, D.; Chadwick, A. V.; Djani, H.; Bousquet, E.; Zhang, W.; et al. Bi2CoO2F4─A Polar, Ferrimagnetic Aurivillius Oxide-Fluoride. *Chem. Mater.* **2022**, *34* (21), 9775-9785. DOI: 10.1021/acs.chemmater.2c02745.

(5) McCabe, E. E.; Free, D. G.; Mendis, B. G.; Higgins, J. S.; Evans, J. S. O. Preparation, Characterization, and Structural Phase Transitions in a New Family of Semiconducting Transition Metal Oxychalcogenides β-La2O2MSe2 (M = Mn, Fe). *Chem. Mater.* **2010**, *22* (22), 6171-6182. DOI: 10.1021/cm1023103.

(6) McCabe, E. E.; Wills, A. S.; Chapon, L.; Manuel, P.; Evans, J. S. O. Structural and magnetic characterization of iron oxyselenides Ce2O2Fe2OSe2 and Nd2O2Fe2OSe2 *Phys. Rev. B* **2014**, *90* (16), 165111. DOI: 10.1103/PhysRevB.90.165111.

(7) Bonfà, P.; Onuorah, I. J.; De Renzi, R. Introduction and a Quick Look at MUESR, the Magnetic Structure and mUon Embedding Site Refinement Suite. In *Proceedings of the 14th International Conference on Muon Spin Rotation, Relaxation and Resonance (?SR2017)*, JPS Conference Proceedings, Vol. 21; Journal of the Physical Society of Japan, 2018.

(8) Isah, M. M.; Dalal, B.; Fung, X.; Mosca, D. F.; Onuorah, I. J.; Scagnoli, V.; Bonfa, P.; Renzi, R. d.; Belik, A. A.; Franchini, C.; et al. Magnetic behavior of 5d1 Re-based double perovskite SrZnReO. *Phys. Rev. B* **2026**, *113*, 104431. DOI: 10.1103/2ngs-7x82.

(9) Steele, A. J.; Baker, P. J.; Lancaster, T.; Pratt, F. L.; Franke, I.; Ghannadzadeh, S.; Goddard, P. A.; Hayes, W.; Prabhakaran, D.; Blundell, S. J. Low-moment magnetism in the double perovskites Ba2MOsO6 (M=Li, Na). *Physical Review B* **2011**, *84* (14), 144416. DOI: 10.1103/PhysRevB.84.144416.
